# Hypoxia Predicts Poor Prognosis in Neuroblastoma Patients and Associates with Biological Mechanisms Involved in Telomerase Activation and Tumor Microenvironment Reprogramming

**DOI:** 10.3390/cancers12092343

**Published:** 2020-08-19

**Authors:** Davide Cangelosi, Martina Morini, Nicolò Zanardi, Angela Rita Sementa, Marco Muselli, Massimo Conte, Alberto Garaventa, Ulrich Pfeffer, Maria Carla Bosco, Luigi Varesio, Alessandra Eva

**Affiliations:** 1 Laboratory of Molecular Biology, IRCCS Istituto Giannina Gaslini, 16147 Genova, Italy; martinamorini@gaslini.org (M.M.); nicolozanardi@gaslini.org (N.Z.); luigivaresio@gaslini.org (L.V.); alessandraeva@gaslini.org (A.E.); 2 Laboratory of Pathology, IRCCS Istituto Giannina Gaslini, 16147 Genova, Italy; angelaritasementa@gaslini.org; 3 Institute of Electronics, Computer and Telecommunication Engineering, Italian National Research Council, 16149 Genova, Italy; marco.muselli@ieiit.cnr.it; 4 Pediatric Oncology Unit, IRCCS Istituto Giannina Gaslini, 16147 Genova, Italy; massimoconte@gaslini.org (M.C.); albertogaraventa@gaslini.org (A.G.); 5 Integrated Oncology Therapies Department, Molecular Pathology, IRCCS Ospedale Policlinico San Martino, 16132 Genova, Italy; ulrich.pfeffer@hsanmartino.it

**Keywords:** neuroblastoma, hypoxia, prognosis, cell immortalization, therapeutic target

## Abstract

The biological and clinical heterogeneity of neuroblastoma (NB) demands novel biomarkers and therapeutic targets in order to drive the most appropriate treatment for each patient. Hypoxia is a condition of low-oxygen tension occurring in poorly vascularized tumor tissues. In this study, we aimed to assess the role of hypoxia in the pathogenesis of NB and at developing a new clinically relevant hypoxia-based predictor of outcome. We analyzed the gene expression profiles of 1882 untreated NB primary tumors collected at diagnosis and belonging to four existing data sets. Analyses took advantage of machine learning methods. We identified NB-hop, a seven-gene hypoxia biomarker, as a predictor of NB patient prognosis, which is able to discriminate between two populations of patients with unfavorable or favorable outcome on a molecular basis. NB-hop retained its prognostic value in a multivariate model adjusted for established risk factors and was able to additionally stratify clinically relevant groups of patients. Tumors with an unfavorable NB-hop expression showed a significant association with telomerase activation and a hypoxic, immunosuppressive, poorly differentiated, and apoptosis-resistant tumor microenvironment. NB-hop defines a new population of NB patients with hypoxic tumors and unfavorable prognosis and it represents a critical factor for the stratification and treatment of NB patients.

## 1. Introduction

Neuroblastoma (NB) is a common extracranial solid tumor of the developing sympathetic nervous system, which accounts for roughly 5% of all diagnosed pediatric cancers [1]. Patients with localized tumors, defined as low or intermediate risk, do not require intensive therapeutic treatment, as, in most cases, the tumor regresses spontaneously. For such patients, surgery is performed when possible and chemotherapy is only considered for symptomatic tumors or for tumor masses growing after surgery [1,2]. On the contrary, patients with disseminated tumors, defined as high-risk, undergo intensive treatment that includes different phases: induction based on chemotherapy at maximally tolerated doses, local treatment with surgery and radiotherapy, consolidation with high dose chemotherapy and peripheral blood stem cells rescue, maintenance based on a differentiating agent (cis-retinoic acid) and, more recently, on immunotherapy targeting the expression of disialoganglioside (GD2) on NB cells. Despite this aggressive treatment, almost 50% of high-risk NB patients are refractory to therapy, relapse, and die [1]. 

Efforts to identify prognostic biomarkers from the genomic interrogation of NB tumors have been made with the aim of improving patient stratification and providing novel therapeutic targets [1,2]. Molecular signatures are becoming increasingly important tools for assisting clinicians in prognosis assessment and therapeutic decisions, because they can be used for accurately predicting patient outcome, relapse, or response to therapy, and also be instrumental for refining patient risk stratification, optimizing treatment, and reducing unnecessary therapy related toxicity [3,4,5,6]. For these purposes, the gene expression profiles of a large number of primary tumor specimens of NB patients have been published in distinct data sets becoming available to the scientific community [6,7,8,9,10,11,12,13]. However, a large-scale expression study of NB tumors has not been previously carried out because different technologies use different proprietary annotations to identify transcripts. The integration of the distinct data sets into one merged data set would enable an unbiased and robust prediction analysis because data set may be split into two large training and test sets. As a consequence, the availability of a large number of gene expression profiles coupled with patient characteristics may enable the stratification of subgroups of patients that are notoriously difficult to analyze with a low number of cases.

The importance of hypoxia in conditioning the aggressiveness of tumors, including NB, is documented by an extensive literature [14,15,16,17]. A variety of techniques have been described to measure intratumor hypoxia including polarographic electrodes, fiber-optic probes, and positron emission tomography, but there is no consensus on the most appropriate approach to use [18]. The identification of an accurate hypoxia predictor may be instrumental for discriminating diagnosis patients who will potentially benefit of an anti-hypoxia therapy, thus preventing treatment-associated damage elicited by unnecessary therapies. We have previously used a biology-driven approach to assess the hypoxic status of NB tumors, consisting in the analysis of the gene expression profile of NB cell lines cultured under hypoxic and normoxic conditions, and identified an 11-probe set hypoxia signature that was able to accurately predict NB patient outcome [19]. However, application of this signature in a large multiplatform study in NB has never been evaluated. 

The tumor microenvironment (TME) is a heterogeneous milieu that is composed by neoplastic, stromal, endothelial, and infiltrating immune cells [20]. The functional interaction among different TME components is critical to determine the development and progression of several types of cancers, including NB [21,22]. Unmasking the altered molecular mechanisms in a hypoxic NB TME may be instrumental to identify novel therapeutic targets and pathways that are involved in NB tumor progression and to design novel personalized therapies for NB patients who have low probability to survive with actual treatment strategies. Hypoxia was reported to strongly affect the TME by altering important biological processes, including tumor cell differentiation, survival, migration, and resistance to therapy, and influencing the nature and function of the immune cell infiltrate [14,23]. However, the molecular mechanisms and biological effectors that are involved in NB hypoxic TME have been only partially elucidated. 

Telomere maintenance mechanisms (TMM) are adopted by tumor cells to prevent telomere shortening and acquire immortality and they represent a malignant hallmark of several cancer cells [24]. Telomerase is a complex ribonucleic reverse transcriptase that is responsible for telomere maintenance by synthesizing telomeric DNA repeats at the 3′ ends of linear chromosomes [24]. The catalytic subunit of the human telomerase reverse transcriptase (TERT) is a key component of the telomerase complex and it is detectable in over 90% of human cancers [25]. Alternative lengthening of telomeres (ALT) is an intra-telomeric recombination mechanism that may be employed by tumor cells to maintain telomere lengthening independently by telomerase activation [24]. Alterations found to be responsible for TMM in tumor cells include TERT rearrangement, somatic mutations of the TERT promoter, ALT, epigenetic changes, and amplification of TERT gene [24]. Despite the large number of publications reporting the critical role of TMM in different diseases, the mechanisms of telomerase regulation remain mostly unknown [26,27]. Ackermann and coworkers have recently shown the unfavorable prognostic impact of TMM in combination with RAS and/or p53 pathway mutations in NB and the correlation of high expression levels of the TERT gene with TMM in a large set of NB specimens [13]. 

In this study, we aimed at assessing the role of hypoxia in the pathogenesis of NB by analyzing the molecular mechanisms and biological effectors that are involved in NB hypoxic TME and at dissecting the prognostic value of a new hypoxia-based predictor in a large multicenter and multiplatform study. Our results show the unfavorable prognostic value of hypoxia in a large number of patients and the ability of hypoxia to additionally stratify clinically relevant groups of NB patients. Furthermore, our results reveal the deregulation of specific biological processes and pathways affecting NB TME. 

## 2. Results

### 2.1. Collection of the Gene Expression Profile of NB Primary Tumors and Patient Characteristics

**Figure 1:**
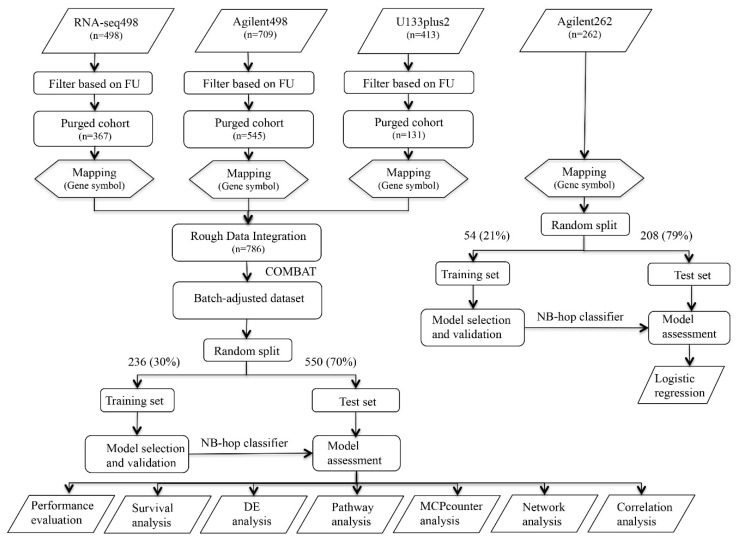
Schematic representation of the procedure used in the study. Workflow of the procedures used to build and test the neuroblastoma (NB)-hop classifier. The gene expression profile of 1620 NB tumors were collected from three different gene expression datasets. Datasets were purged of incomplete and unreliable samples. COMBAT adjusted the data for batch effect removal. The resulting dataset of patients was divided into training and test sets. LibSVM was used to build the NB-hop classifier in the batch-adjusted and Agilent262 data sets. The performance of the NB-hop classifier was then assessed in the test set. Survival analysis evaluated the clinical relevance of the NB-hop classifier. Differential expression analysis (DEA) and pathway analysis explored the molecular mechanisms altered between favorable and unfavorable NB-hop tumors. Microenvironment cell populations (MCP)-counter method estimated the abundance of immune and stromal cell populations. Network analysis assessed the functional association among genes. Correlation analysis estimated the strength of relationship between the expression of two genes. An additional data set composed by 262 gene expression profiles from untreated primary NB tumors coupled with patient status was used for investigating the link between hypoxia and TMM and/or telomerase activity. FU: Follow-up. NB-hop: Neuroblastoma hypoxia outcome predictor. SVM: Support vector machine. DEA: Differential expression analysis. MCP-counter: Microenvironment cell populations-counter.

Analyses were carried out using the 11-probe set signature to assess the role of hypoxia in the pathogenesis of NB and to dissect the prognostic value of a new hypoxia-based predictor in a large multicenter and multiplatform study [19]. To these aims, we collected the gene expression profile of 1882 NB tumor specimens covering the entire spectrum of the disease included into four publicly available data sets (RNA-seq498, Affymetrix413, Agilent709, and Agilent262) [6,7,8,9,10,11,12,13]. Figure 1 shows the schematic representation of the analyses carried out in the present study. 

The relative percentage of patient outcome was comparable among the four data sets. Table 1 summarizes platform information, clinical, and molecular characteristics of patients. 

Age at diagnosis, international NB staging system (INSS) stage, MYCN status, event overall, and event-free data were available for all data sets. Patient follow-up was available for RNA-seq498, Affymetrix413, and Agilent709 data sets, but not for Agilent262, whereas telomere maintenance, ALT, documented regression, TERT rearrangement, ATRX mutation, RAS, and p53 mutation data were only available for the Agilent262 data set.

### 2.2. Integration of Gene Expression Profiles of NB Primary Tumors Using the COMBAT Batch-Effect Removal Method

The gene expression profiles of RNA-seq498, Agilent709, and Affymetrix413 data sets were integrated into a single data set to achieve a large-scale genomic data analysis. 577 patients were filtered out from the analysis either because of missing information about outcome or of follow-up shorter than five years (Figure 1). Furthermore, 257 patients that were profiled with both Illumina and Agilent technologies were removed from the Agilent709 data set to have independent data sets. Proprietary identifiers of each platform were mapped into gene symbols for comparability. The new merged and filtered data set comprised 786 patients that included 288 patients from Agilent709, 367 from RNA-seq498 and 131 from Affymetrix413.

It is known that the batch effect may be introduced when data sets from different gene expression platforms are integrated [28]. Batch effect can be estimated using principal variance component analysis (PVCA). PVCA uses the weighted average proportion variance (WAPV) to estimate the magnitude of any source of variability using biological, clinical, and batch variables [28]. Thus, the presence of a potential batch effect in the merged data set was assessed by PVCA. The PVCA results showed a WAPV of platform of 0.79, indicating that data integration introduced a measurable batch effect (Figure S1). Several computational methods have been proposed to remove batch effect from the data [28]. COMBAT is a well-known method to remove batch effect in the data applying an empirical bayes approach [28]. The application of the COMBAT technique to the gene expression profiles of RNA-seq498, Agilent709 and Affymetrix413 data sets removed the batch effect introduced by integrating the data from three different platforms (WAPV of Platform = 0.0; Figure S2). Furthermore, analysis evidenced the variance explained by the biological and clinical variables (WAPV > 0, Figure S2). The 786 gene expression profiles of the batch-adjusted data set are available in Table S1.

The expression profiles of 236 randomly selected tumors out of 786 (30%) served to build a classifier and the profiles of the remaining 550 tumors (70%) were used to test its prognostic value in a validation data set. The clinical and molecular characteristics of patients in the training and test sets are summarized in Table S2 and listed in Table S3. 

### 2.3. Identification of a New Multiplatform Hypoxia Biomarker

We have previously used a biology-driven approach in order to assess the hypoxic status of NB cells identifying a 11 probe set signature that was able to accurately predict NB patient outcome [19]. This signature could not be used in the present study because the probe identifiers used were specific of the Affymetrix U133 plus 2.0 gene expression platform. Hence, we refined the signature by mapping the probe sets into gene symbols to obtain a multi-platform biomarker. 9 out of the 11 probe sets were annotated with a gene symbol, whereas two were not associated with a gene symbol and were excluded from subsequent analysis. Seven out of nine gene symbols were unique. Therefore, a new seven-gene biomarker named NB-hop (NB-hypoxia outcome prediction) was defined. Table 2 lists the main characteristics of NB-hop genes. 

These genes encode for proteins that are involved in metabolic response to hypoxia. Univariate analysis of overall survival in the batch-adjusted training set based on the NB-hop genes showed that high expression of PGK1, PDK1, MTFP1, and FAM162a genes was associated with a significantly higher risk of death (hazard ratio (HR) > 1 and *p*-value < 0.05; Table 2), while a high expression of ALDOC was associated with a lower risk of death (HR < 1 and *p*-value < 0.05; Table 2). 

### 2.4. Generation and Validation of a NB-hop Classifier for Predicting NB Patient Prognosis

**Figure 2:**
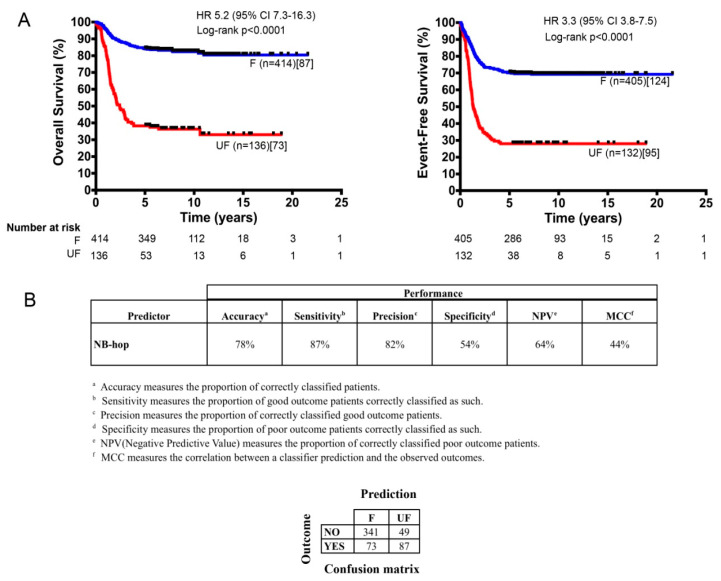
Kaplan–Meier estimates of Overall survival (OS) and Event-free survival (EFS) and prediction performances of the NB-hop classifier (**A**). The OS (left plot) and the EFS (right plot) of the two populations of NB patients predicted by the NB-hop classifier in the test cohort (*n* = 550) are shown. The classifier was built from the expression of NB-hop genes and patient outcome in the training set using the LibSVM library and leave one-out cross validation (LOOCV) technique. Blue and red curves represent the F and the UF classifications, respectively. Curves were compared by log-rank test. Log rank *p* value, number of patients classified by NB-hop (brackets), and number of deaths or events in each group of patients (square brackets) are reported. The number of patients at risk is displayed under the Kaplan–Meier plots. Each plot reports the hazard ratio (HR) and 95% of confidence interval (95% CI). (**B**) The NB-hop classifier prediction performances, measured by accuracy, sensitivity, precision, specificity, NPV, and MCC are shown. A brief description of the performance measures and the confusion matrix appear under the table. OS: Overall survival; EFS: Event-free survival; F: Favorable; UF: Unfavorable; HR: hazard ratio; CI: confidence interval; NPV: negative predictive value; MCC: Matthew’s correlation coefficient.

The classifier was built from the expression of NB-hop genes and patient outcome in the training set using the LibSVM library and the leave one-out cross validation (LOOCV) technique (Section 4). The predictive power of the NB-hop classifier was then estimated in the test set. NB-hop classifier predicted 414 out of 550 patients (75%) at favorable prognosis (F) and 136 out of 550 patients (25%) at unfavorable prognosis (UF). Moreover, it was able to stratify patients into subgroups that had a significantly different overall survival (OS) and event-free survival (EFS) (OS: HR 5.2 95% confidence interval (CI) 7.3–16.3 and EFS: HR 3.3 95% CI 3.8–7.5, both *p* < 0.0001; Figure 2A). 

Our classifier obtained a significant overall performance of 44% of Matthew’s correlation coefficient (MCC) (78% of Accuracy) in the test set (Figure 2B and Table S3). For comparison, we trained and tested four alternative machine learning algorithms on the batch-adjusted data set. The performance of each of these methods was lower than that of libSVM (MCC < 44%, Table S4). To assess the significance of the NB-hop classifier performance, we applied the ConfusionMatrix function that was implemented in the Caret R Package [29] to NB-hop prediction and event overall in the batch-adjusted test set. We found that the no information rate was 0.709 and the difference of accuracy between NB-hop classifier and no information rate was significant (*p* value < 0.0001). These findings support our conclusion that the NB-hop classifier is an accurate predictor of NB patients’ outcome. 

**Figure 3:**
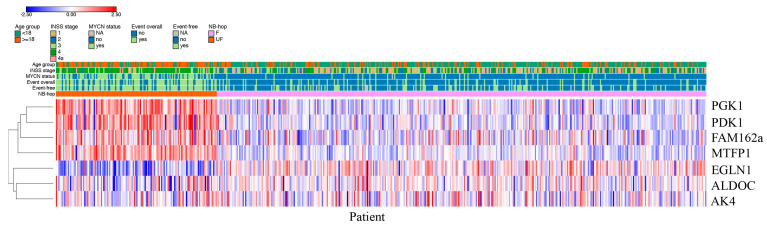
Heat map based on NB-hop gene expression in the batch-adjusted test set. The expression values for each gene belonging to the NB-hop signature (rows) have been scaled and are represented by pseudo-colors in the heat map. Red color corresponds to high level of expression and blue color corresponds to low level of expression. Patients (columns) are divided into two groups according to NB-hop predictions. Indicated on top of the heat map are age groups, INSS stage, MYCN status, event overall, event-free and NB-hop prediction with the relative color legend. The expression value of the seven NB-hop genes was grouped by hierarchical clustering. The hierarchical clustering dendogram is shown on the left. F: Favorable. UF: Unfavorable. NB-hop: neuroblastoma hypoxia outcome predictor.

Patients predicted as UF by NB-hop had a clear different expression profile with respect to those predicted as F NB-hop, demonstrating that NB-hop was able to distinguish two groups of patients at the gene expression level (Figure 3). 

HIF-1a and HIF-2a are hypoxia inducible factor α-subunits that mediate the cellular response to hypoxia [16]. We compared the distribution of HIF-1a and EPAS1/HIF-2a mRNA expression of NB patients grouped by NB-hop prediction in batch-adjusted test set. Box plots displayed in Figure S3 show a significant up-regulation of HIF-1a and a significant down-regulation of EPAS1/HIF-2a expression in the group of UF NB-hop tumors (*p* < 0.0001). These data indicate that UF NB-hop tumors are more hypoxic than F NB-hop tumors. We also correlated the HIF-1a gene expression and that of each NB-hop marker by Pearson correlation. We found a significant correlation between HIF1a and NB-hop markers (*p* < 0.05). 

The prognostic power of the NB-hop classifier was compared with that of established markers utilizing NB-hop classifier predictions (UF vs. F), INSS stage (4 vs. 1, 2, 3, 4 s), age at diagnosis (age ≥ 18 months vs. < 18 months), and MYCN status (amplified vs. single copy) in the test set. UF NB-hop, advanced stages 4, age ≥ 18 months, and MYCN amplification were significantly associated with a higher risk of death or undergoing an event according to a univariate analysis (HR > 1 and *p* < 0.0001; Table 3).

Importantly, the NB-hop classifier maintained a significant prognostic effect in the model adjusted for these clinical covariates in multivariate analysis (OS: HR 1.8 95% CI 1.2–2.6, *p* = 0.004 and EFS: HR 1.7 95% CI 1.2–2.5, *p* = 0.001; Table 3). We concluded that NB-hop is an independent prognostic biomarker of unfavorable prognosis in NB.

**Figure 4:**
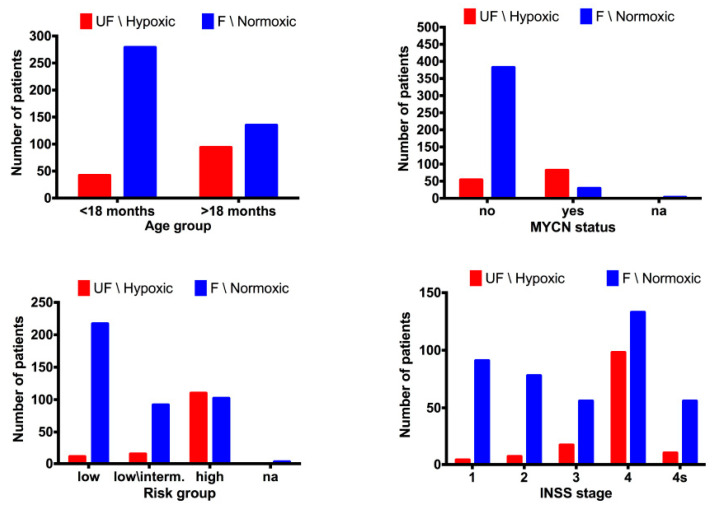
Bar plots of the distribution of NB-hop predictions in the batch-adjusted test set. The bar plots shows the number of patients with unfavorable prognosis (UF) NB-hop (red) and F NB-hop (blue) on the y-axis and one of the reference variables (age at diagnosis, INSS stage, MYCN status, and risk group) on the x-axis. Age at diagnosis was split into two groups, one >18 months and the other <18 months. Risk group was divided into low, low/intermediate, and high risk on the basis of International NB risk group (INRG) pre-treatment risk stratification schema. Na stands for not accessible value. NB-hop prediction labels are displayed on top of each bar plot.

We evaluated the distribution of the two populations of patients with F and UF prognosis in the subsets defined by age at diagnosis, INSS stage, MYCN status, and risk group. The number of patients predicted with UF NB-hop was greater than zero in all subsets, but it was higher in patients with age greater than 18 months, INSS stage 4, amplified MYCN, and high-risk disease (Figure 4). 

These results indicate that UF patients are associated with unfavorable clinical characteristics and suggest that NB-hop may additionally stratify clinical groups of patients. We assessed the stratification of sub-cohorts defined by known prognostic markers in order to test this hypothesis (Table 4).

The NB-hop classifier significantly stratified patients with tumor stage 1, 2, 3, and 4, patients with age < 18 months, patients with age > 18 months, and patients with not amplified MYCN tumor (*p* < 0.05). The NB-hop classifier was not able to significantly stratify patients with amplified MYCN or stage 4s tumors (*p* > 0.05). 

**Figure 5:**
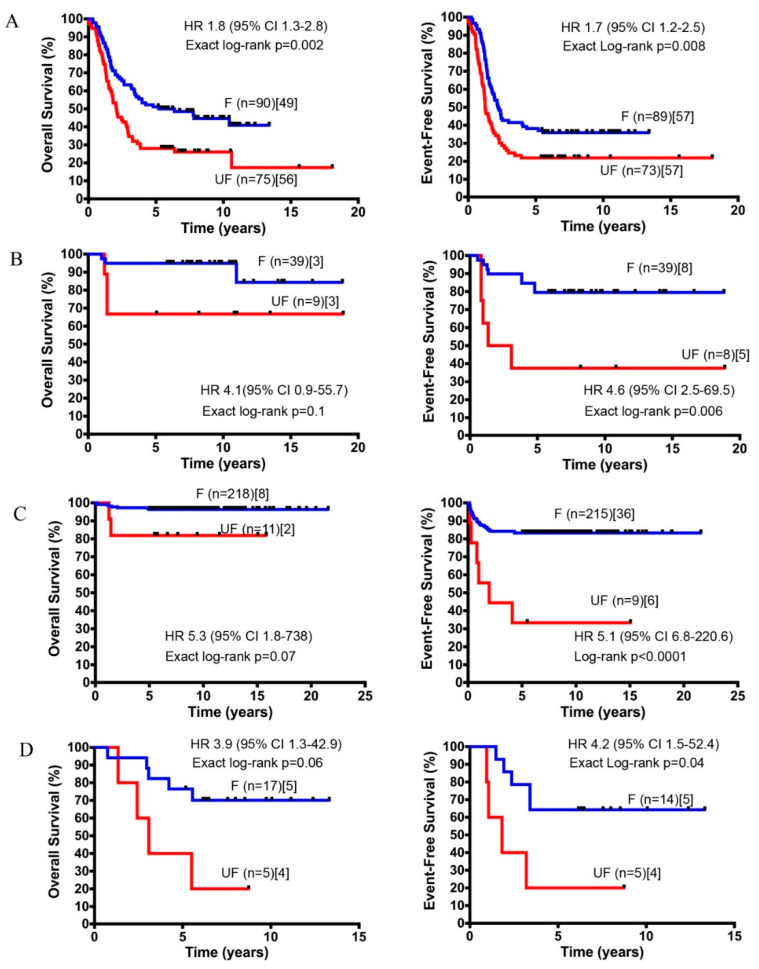
OS and EFS of clinically relevant sub-groups of patients stratified by the NB-hop classifier. Kaplan-Meier curves show OS and EFS of NB patients in the test set predicted by NB-hop classifier. Plots are relative to (**A**) high-risk patients, stage 4, and age > 18 months, (**B**) intermediate-risk patients, stage 4, age < 18 months, and with not amplified MYCN tumors, (**C**) low-risk patients with stages 1, 2, 4s and no MYCN amplification tumor, (**D**) intermediate-risk patients, age > 18 months, stage 3 with not amplified MYCN tumors. Plots are entitled with the characteristics of the patients in the sub-population. F NB-hop (blue) and UF (red) curves were compared by approximate log-rank test or exact log-rank test when approximate log-rank *p* value <0.0001 or >0.0001, respectively. Each plot reports the HR and 95% CI. Number of patients classified as F or UF (brackets), and the number of patients who succumbed to disease or underwent an event (square brackets) are reported. OS: Overall survival; EFS: Event-free survival; HR: hazard ratio; CI: confidence interval; F: favorable; UF: Unfavorable.

Next, the prognostic value of our predictor was assessed in additional clinically relevant subgroups of patients defined by combination of established prognostic markers. The group of high-risk patients older than 18 months with stage 4 tumors is a group traditionally difficult to stratify [1]. NB-hop classifier identified two subgroups of patients with significantly different OS and EFS (OS: HR 1.8 95% CI 1.3–2.8, *p* = 0.002 and EFS: HR 1.7 95% CI 1.2–2.5, *p* = 0.008; Figure 5A). 

The NB-hop classifier achieved a significant prediction performance of 21% MCC (Fisher *p*-value = 0.009). In the subgroup of low and intermediate-risk patients with stage 4 tumors lacking MYCN amplification and age < 18 months, NB-hop classifier identified two groups of patients with significant different EFS (EFS: HR 4.6 95% CI 2.5–69.5, *p* = 0.006; Figure 5B), but did not significantly discriminate patients who died from disease in this subgroup (OS: HR 4.1 95% CI 0.9–55.7, *p* = 0.1; Figure 5B). 

In the subset of patients with stage 1, 2, 4s tumors lacking MYCN amplification, who are notoriously at low-risk of dying of disease [2], our classifier was able to discriminate patients who underwent an event from those who did not (EFS: HR 5.1 95% CI 6.8–220.6, *p* < 0.0001 Figure 5C). On the contrary, NB-hop was not able to significantly stratify patients who died, even though we observed a positive trend (OS: *p* > 0.05, Figure 5C). Patients that were older than 18 months with stage 3 and not amplified MYCN tumor constitute a group of localized tumors at intermediate risk, whose stratification remains a challenge [30]. NB-hop classifier was able to additionally stratify this population of patients (OS: HR 3.9 95% CI 1.3–42.9 and EFS: HR 4.2 95% CI 1.5–52.4, *p* ≤ 0.05 Figure 5D).

These findings highlight the ability of NB-hop to stratify NB patients that are difficult to stratify with actual risk assignment, indicating the potential prognostic significance of this classifier in NB. 

### 2.5. Comparative Transcriptome Analysis of Primary Tumor Specimens between NB-hop Unfavorable and Favorable Patients

Microarray and RNA-seq platforms provide expression quantification of several transcripts in parallel from a sample. We compared the tumor transcription profile between F and UF populations of NB patients predicted by NB-hop classifier in the test set in order to investigate the key molecular mechanisms altered in NB hypoxic TME. Expression differences ≥1.5 or ≤−1.5 and Benjamini–Hochberg q-value ≤0.05 were considered to be statistically significant. Using these selection criteria, we identified 2377 differentially expressed genes (DEGs) (Table S5). Among them, 926 were up-regulated and 1451 were down-regulated in UF NB-hop with respect to F NB-hop tumors. 

Pathway analysis is a well-known bioinformatic tool that is used to explore the biological processes and pathways associated with a list of differentially expressed genes using curated ontologies [31]. The list of DEGs was analyzed by the STRING-DB software using Gene ontology (GO), KEGG, and REACTOME ontologies (see Materials and Methods). Pathway analysis of up and down-regulated genes identified 546 and 705 significantly enriched biological processes and pathways in UF NB-hop, respectively. Table S6 reports the complete list of significant processes and pathways for every analysis. Table 5 presents a selection of significantly enriched functional terms. 

Up-regulated genes were mainly involved in the cellular response to hypoxia, telomere maintenance, chromatin remodeling, DNA damage response, P53 mediated cell cycle arrest, cellular senescence, cell cycle, and proliferation. Down-regulated genes were mainly involved in immune response, cell differentiation, motility, inflammation response, cell death, and angiogenesis. These results showed that patients with UF NB-hop tumor are characterized by a hypoxic, immune suppressive, poorly differentiated, and apoptosis-resistant TME. 

Table 6 lists DEGs whose role has been previously reported in NB.

The up- or down-regulation of these genes have been previously associated with an unfavorable prognosis of NB patients, in accordance with our results. Among the up-regulated genes, several were involved in glycolysis (GAPDH, HK2, PGK1, PKM, LDHA, LDHB, and SLCO4A1), pH regulation (SLC16A1), and homeostasis (PDK1), indicating a metabolic reprogramming typical of hypoxic cells. In addition, a prominent set of up-regulated genes coding for proteins with a primary role in telomere maintenance (FEN1, PCNA, and TERT), DNA damage response (BRCA1, BRCA2, CHEK1, CHEK2, and TPX2), P53 mediated cell cycle arrest (CDK1, CDK2, and TP53), and proliferation (AURKA, ERBB4, LIN28B, LMO3, MYCN, ODC1, and RAN). On the contrary, we demonstrated the down-regulation of genes coding for proteins that are involved in immune responses (CADM1, CCL19, CCL2, CCR7, CD226, and CXCL12), cell differentiation (CAMTA1, DNER, NTRK1, and PTN), cell motility and invasion (CD44, CD9, CDH1, ERBB3, L1CAM, NRP1, and SRCIN1), and angiogenesis (EPAS1). We also found the down-regulation of genes coding for proteins that are involved in chromatin remodeling (ARID1A and CHD5) and of the MYC gene and the up-regulation of genes involved in cell differentiation (ALK, PROM1, and RET), and coding for proteins that are involved in apoptosis and cell death (BIRC5 and TWIST1). 

We performed a network analysis using STRING-DB software in order to assess the biological connection between HIF-1a and genes reported in Table 6 [89]. The resulting network that is shown in Figure S4 displayed significantly more interactions than expected for a random set of proteins of similar size drawn from the genome (PPI enrichment *p*-value < 1.0 × 10^−16^). Our findings establish a connection between HIF-1a and genes reported in Table 6 and indicate that those protein-coding genes are biologically connected as a group. 

### 2.6. Correlating Immune Markers with NB-Hop Prediction and HIF1 Signature

**Figure 6:**
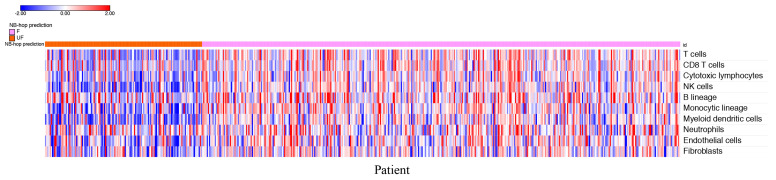
Heat map of the cell abundance scores in the batch-adjusted test set. The MCP-counter score values for each cell type (rows) have been scaled and are represented by pseudo-colors in the heat map. Red color corresponds to high level of the score and blue color corresponds to low level of the score. NB patients belonging to the batch-adjusted test set (columns) are divided into two groups according to NB-hop predictions. The color key is displayed on the top left part of the plot. MCP: Microenvironment cell populations; F: Favorable; UF: Unfavorable; NB-hop: Neuroblastoma hypoxia outcome predictor.

The estimation of the abundance of immune and stromal cell populations in the TME may uncover their role in tumor development/progression [90]. Differential expression analysis provides useful information regarding deregulated genes, but it is not suitable for estimating the abundance of different tumor-infiltrating cells. For this purpose, we applied the microenvironment cell populations (MCP)-counter method using the expression profile of NB tumors in the batch-adjusted test set. MCP-counter is a very well-known and widely used bioinformatic tool. Furthermore, transcriptome-based cell-type quantification methods for immuno-oncology are valuable tools with several successful applications [91]. MCP-counter returned an abundance score for each cell type and tumor sample in the test set. Figure 6 depicts the heat map of the scores for each patient grouped by NB-hop prediction. 

The heat map shows a clear tendency to a lower abundance of T cells, CD8+ T cells, NK cells, cytotoxic lymphocytes, B cell lineage, monocytic lineage cells, myeloid dendritic cells, endothelial cells, and fibroblasts in patients with UF NB-hop tumors than in F NB-hop tumors, suggesting the association between an immunosuppressive TME and UF NB-hop tumors. 

We carried out additional analyses to provide more links between MCP-counter analysis and hypoxia. For each cell type MCP-counter defines a set of characteristic genes that we refer to as MCP-counter markers [90]. Overlap between MCP-counter markers and genes in the HIF-1a interaction network may provide relevant evidence of a direct involvement of HIF-1a in the regulation of immune or non-immune cell population activity. To this end, we assessed the overlap between the list of MCP-counter markers and the list of genes in the HIF-1a interaction network. BioGRID is a public curated gene interaction repository [92]. Three-hundred and eighty-nine HIF-1a interactor genes were retrieved from the BioGRID repository. The resulting HIF-1a interaction network is shown in Figure S5. Venn diagram showed that none of the MCP-counter markers were in the HIF-1a interaction network. Network analysis is a valuable tool for assessing the functional interaction among a set of genes. We performed a network analysis for HIF-1a and MCP-counter markers using the STRING-DB software [89]. Interestingly, we found that HIF-1a was functionally associated with MCP-counter markers (Figure S6). These results showed a significant functional association among HIF-1a and MCP-counter markers, raising the question of the possible correlation between HIF-1a and MCP-counter markers in NB. Thus, we carried out a correlation between the expression of HIF-1a and that of each MCP-counter marker in the batch-adjusted test set using Pearson’s correlation method. The results reported in Table S7 showed that high HIF-1a expression is negatively correlated with most of the markers of T cells, CD8 T cells, cytotoxic lymphocytes, NK cells, myeloid dendritic cells cell types (Pearson r < 0 and *p* value < 0.05, Table S7), and positively correlated with most of the makers of monocytic lineage, neutrophils, endothelial cells and fibroblasts (Pearson r > 0 and *p* value < 0.05, Table S7). Within the set of significantly correlated immune markers in Table S7, we found that MAL, BANK1, CXCR2, and KCNJ15 genes have been previously reported to play a tumor suppressive role in different types of cancer [93,94,95,96]. These findings support the results shown in Figure 6. 

The presence of specific immune regulatory cell populations is associated with poor outcome in different types of cancer, including NB [97]. Tumor infiltrating macrophages (TAMs) often display an immunosuppressive M2-phenotype in aggressive NB tumors [97]. Gene expression analysis of primary human NB tumors without MYCN amplification has revealed that high-level expression of TAM-specific genes, including CD14, CD16, IL6, IL6R, and TGFB1, was associated with poor five-year event-free survival [98]. We assessed the correlation between the expression of HIF-1a and that of TAM-specific genes in the batch-adjusted test set with not amplified MYCN tumors in order to find additional associations between hypoxia and immune suppressive TME in NB. The results reported in Table S8 showed a significant positive correlation between HIF-1a and CD14, IL6, and IL6R, while no significant correlation was found between HIF-1a and TGFB1. CD16/FCGR3A was absent in the batch-adjusted data set and it was excluded from the analysis. These findings suggest that hypoxia is associated with the presence of TAMs in NB without MYCN amplification supporting our conclusion that hypoxia is an unfavorable prognostic marker and is associated with an immune suppressive TME in NB.

One of the main mechanisms exploited by tumors for immune system escape is modulating immune checkpoints, inhibitory pathways that physiologically maintain self-tolerance, and limit the duration and amplitude of immune responses [99]. We focused on the immune checkpoint ligands PD-L1 (CD274), PD-L2 (PDCD1LG2), and B7-H3 (CD276), which are recognized by inhibitory receptors that are expressed by T and NK cytolytic immune effectors [99]. Because no PD-L2 gene expression was detected in the batch-adjusted data set, a comparison between UF and F NB-hop tumors was carried out for B7-H3 and PD-L1 expression. The results presented in Figure S7 showed that B7-H3 was significantly up-regulated, whereas PD-L1 was significantly down-regulated in UF NB-hop tumors, thus indicating a differential mRNA expression modulation of these immune checkpoints in NB tumors.

### 2.7. Validation of the NB-hop Classifier for Predicting TERT Gene Over-expression in an Independent Cohort of NB Patients

We used the gene expression profiles of 262 untreated primary NB tumors (Agilent262), for the majority of which we had information on the TMM activation in addition to other patient clinical and molecular data, such as age at diagnosis, ALT activation, event overall, event-free, TERT rearrangement, INSS stage, MYCN status, p53/RAS gene mutations, and spontaneous regression, in order to evaluate the association between hypoxia and TMM in NB [13]. Patients included in the data set did not overlap with the 786 patients in the batch-adjusted data set. Similarly to that described for the batch-adjusted data set, a classifier based on the expression of the 7 NB-hop genes and patient outcome was built in the training set (54 patients equal to 21%) using the LibSVM library and LOOCV technique. The predictive power of the NB-hop classifier was validated in the test set (208 patients equal to 79%). NB-hop classifier predicted a favorable prognosis for 138 out of 208 patients (66%) and an unfavorable prognosis for 70 out of 208 patients (33%). The clinical and molecular characteristics and NB-hop predictions of patients are reported in Table S9. 

**Figure 7:**
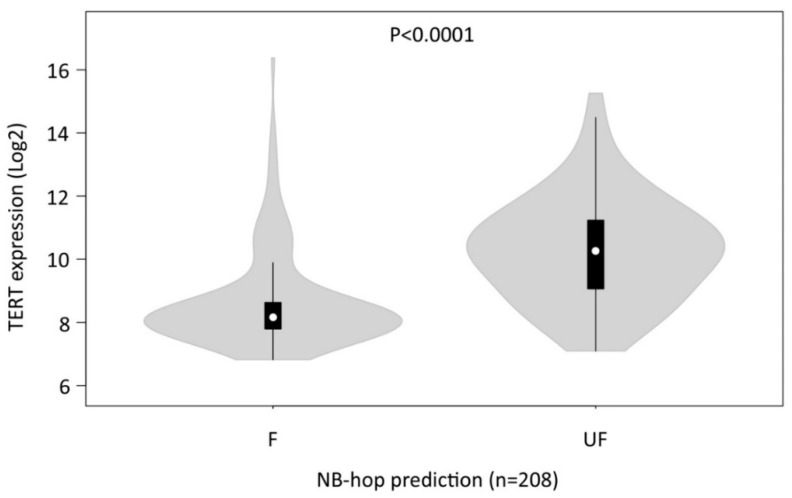
Distribution of telomerase reverse transcriptase (TERT) mRNA expression in NB patients grouped by NB-hop prediction in the Agilent262 test set. Violin plot show the distribution of TERT expression in mRNA profiles of NB patients grouped by NB-hop prediction. Data are relative to Agilent262 test set (*n* = 208). Significance of the expression differences between F and UF NB-hop groups of patients was assessed by unpaired *t* test. *p*-value is reported on the top. F: Favorable; UF: Unfavorable; NB: Neuroblastoma.

We, then, analyzed the distribution of TERT mRNA expression in the profiles of patients belonging to the Agilent262 test set that was grouped by NB-hop predictions. We found that TERT mRNA expression was significantly elevated in patients with UF prognosis with respect to those predicted with F prognosis (*p* < 0.00001, Figure 7). 

These findings confirmed the association between hypoxia and telomerase activity/expression, raising the question of the importance of hypoxia for TMM in NB. 

### 2.8. Univariate Regression Analysis for Assessing the Association between Hypoxia and Telomere Maintenance Mechanisms in NB

We investigated the association between hypoxia, TMM, and other available clinical and molecular prognostic factors for NB [13] in the Agilent262 test set by univariate logistic regression analysis. This analysis showed that patients with UF expression of the NB-hop biomarker had higher odds of having an age > 18 months, of undergoing an event or relapse/progression, INSS 4 stage tumor, MYCN amplification, p53/RAS gene mutations, or TMM (odd ratio > 1 and *p*-value < 0.05, Table 7). The association between NB-hop and TMM is still significant even excluding amplified MYCN tumors from the data set (Odd ratio > 1 *p* < 0.05). 

**Figure 8:**
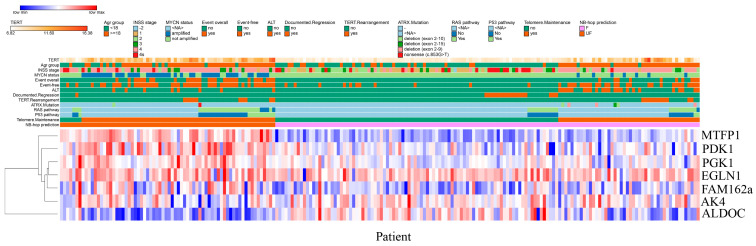
Heat map visualization of NB-hop gene expression in the Agilent262 test set. The expression values for each gene belonging to the NB-hop signature (rows) have been scaled and are represented by pseudo-colors in the heat map. Red color corresponds to high level of expression and blue color corresponds to low level of expression. NB patients (columns) belonging to the Agilent262 test set are divided into two groups according to NB-hop predictions. Age groups, INSS stage, MYCN status, event overall, event-free, TMM, ALT, p53/RAS gene mutations and NB-hop prediction are indicated on the top of the heat map with the relative color legend. The expression value of the 7 NB-hop genes was grouped by hierarchical clustering. The hierarchical clustering dendogram is shown on the left. NB-hop: neuroblastoma hypoxia outcome predictor; TMM: Telomere maintenance mechanisms; ALT: Alternative lengthening of telomeres; F: Favorable; UF: Unfavorable.

On the contrary, patients with documented tumor regression were associated with a lower odd of UF NB-hop prognosis (odd ratio < 1 and *p*-value < 0.05, Table 7). No significant odd ratio was found between NB-hop prediction and ALT or TERT rearrangements, which indicated that NB-hop prediction is not related to these factors (*p*-value > 0.05, Table 7). Heat map visualization of NB-hop gene expression and established prognostic factors for NB showed that UF NB-hop patients had a clear different expression profile with respect to F NB-hop ones, confirming that NB-hop was able to distinguish two distinct groups of patients with different prognosis at the gene expression level (Figure 8).

Furthermore, heat map graphically showed the association between the UF NB-hop subgroup and the worse clinical and molecular characteristic of NB patients, in accordance with the univariate regression analysis. These findings provide an indication that hypoxia is associated with telomerase activity, thus representing a potential critical factor for the classification and treatment of NB patients.

## 3. Discussion

Novel prognostic markers and therapeutic targets are urgently needed in the clinical settings to improve prognosis of NB patient and design more effective treatments and better-tailored therapies addressing NB heterogeneity [1]. Here, we propose a new seven-gene hypoxia biomarker, referred to as NB-hop, as an unfavorable prognostic marker for NB patients.

The NB-hop biomarker was defined by gene expression analysis carried out on 1620 tumor specimens belonging to three available data sets, whose expression was measured by three different gene expression platforms, which included Agilent, Illumina, and Affymetrix technologies. The RNA-seq498, Agilent709, and Affymetrix413 data sets were homogeneous on the basis of patient clinical and molecular data and they were used for dissecting the prognostic value of the new NB-hop hypoxia biomarker. Analysis of the gene expression profile of NB primary tumors to find prognostic factors is an active research field, and several prognostic signatures have been reported [5,6,100]. However, a large-scale expression study of NB tumors has not been previously carried out because different technologies used different proprietary annotations to identify transcripts. Meta-analysis is traditionally used in retrospective studies to overcome this problem [28]. However, meta-analysis must be replicated independently in each data set, limiting the statistical power of the study [28]. Here, we used a novel approach that is based on the application of the batch effect removal methods [28] for integrating the RNA-seq498, Agilent709, and Affymetrix413 data sets [28]. Data integration raises the question of the presence of the so-called batch effect [28]. Our analysis evidenced that the simple integration of the NB datasets introduces a measurable batch effect, which could hinder subsequent analysis. Several batch effect removal methods have been described in the literature [28]. In the present study, we used COMBAT, a well-known technique to remove batch effect for data integration [28]. The application of this method to integrate gene expression data of NB tumor samples profiled by different platforms allowed us to build up one single multiplatform and multicenter cohort of 786 patients, with at least five years of follow-up, representing the largest data set described so far in a gene expression study of NB patients. 

The expression profiles of 30% NB tumor samples served to build a classifier and the profiles of the remaining 70% were used to test its prognostic value in a validation data set. Split a data set into a training set and a test set is a standard machine learning procedure for computing an unbiased estimation of the performances of a classifier. In the test set, the NB-hop classifier distinguished two groups of patients at the gene expression level. The UF NB-hop group was composed by 25% of samples and was referred as the group with hypoxic tumor and unfavorable prognosis. F NB-hop was composed by 75% of samples and referred as the group with normoxic tumor and favorable prognosis. OS and EFS were significantly lower for UF NB-hop respect to F NB-hop patients. UF NB-hop tumors were associated with unfavorable clinical characteristics including age > 18 months, INSS stage 4, amplified MYCN, or high-risk disease, which confirmed the unfavorable prognosis of UF NB-hop patients. We demonstrated that NB-hop retains its independent prognostic effect in multivariable analysis that included age at diagnosis, INSS stage, and MYCN status, which are considered the strongest prognostic clinical and molecular variables for prediction of OS and EFS in NB [2]. Analysis was focused on established risk factors in NB, such as age at diagnosis, MYCN status, and INSS stage, whose data were available in the original data sets, but not on other known risk factors, such as chromosomal aberration, ploidy, grade of differentiation, or histological category [2], because these data were not reported in the original publications. 

The clinical value of novel prognostic factors is often evaluated in selected groups of patients defined by combination of established NB risk factors [5,6,100]. In this study, we assessed the prognostic value of our biomarker in clinically relevant groups of NB patients whose stratification was not previously reported. The groups included patients with metastatic (INSS stage 4) disease and age > 18 months, patients with age < 18 months, metastatic disease (INSS stage 4) and not amplified MYCN, and patients with localized (INSS stage 1, 2) or metastatic (INSS stage 4s) tumors with not amplified MYCN, and patients with age > 18 months, stage 3 and not amplified MYCN that are classified as high, intermediate, and low risk according to INRG schema [1,2,30]. We demonstrated that NB-hop additionally stratified these groups of patients, making of NB-hop a new potentially eligible biomarkers for inclusion in upcoming NB pre-treatment risk schema.

NB-hop classifier is a valuable tool with potential direct application in the clinical management of children that are affected by NB. A classifier may be used to discriminate at diagnosis patients with hypoxic and unfavorable tumor from those with normoxic and favorable one. For patients with hypoxic and unfavorable tumor, classifier may be used for additionally refining patient risk stratification, optimizing treatment, and reducing unnecessary therapy related toxicity that remain among the most relevant open questions in the clinical discussion [3,4,5,6]. Nowadays, the inclusion of new prognostic biomarkers into the clinical practice is limited, probably because of the relatively small number of patients used so far in similar studies and the consequent limited reproducibility of the analyses. Our classifier is robust, because it was trained and tested using a large number of gene expression profiles measured with different platforms. Still, a potential limitation of the applicability of our classifier may depend on NB tumor samples that are available for gene expression profile necessary to determine the hypoxic status of these tumors. In fact, it is often difficult to surgically obtain tumor samples from young children. 

NB-hop genes are known from the literature to be modulated by hypoxia and encode proteins that are involved in key biological processes associated with the response to hypoxia [3,17,101,102,103,104,105,106,107,108,109]. Expression of these genes has been previously reported to represent a marker of hypoxia in NB cell lines [3,102,105] and tumors [102], and to correlate with NB bone marrow metastases and poor patient outcome [3,101]. The expression of NB-hop genes correlates with HIF-1a expression, which is one of the most important regulators of the cellular response to hypoxia [16]. 

Low oxygen levels are known to induce remarkable transcriptional changes that affect the entire TME [20]. Several scientific publications have evidenced the effects of NB cells adaptation to the hypoxic stress in terms of cell cycle arrest, cell differentiation, cellular stemness, resistance to cell death, genomic instability, cellular motility, invasiveness, and proliferation [14,23,110]. However, the molecular mechanisms mediating the NB tumor response to the hypoxic TME in NB have been only partially understood [23]. Our study is the first one assessing the hypoxia-deregulated transcription program in a large set of NB tumors.

A comparison of the expression profiles between UF and F NB-hop tumors showed the significant modulation of a large number of genes. Two distinct types of analysis were used in order to identify the pathways and cellular processes associated with the expression of these genes. Firstly, we utilized a pathway analysis based on three public ontologies. This approach was instrumental to identify the most affected biological processes and pathways associated with UF NB-hop tumors. Secondly, we performed a bibliographic search to identify the genes whose role in the pathogenesis and prognosis of NB had already been reported. Our findings evidence that the UF NB-hop tumors display up-regulation of genes that are known to be induced by hypoxia and to be involved in the glycolytic pathway, pH regulation, and homeostasis [3,5,32,33,34,35]. Importantly, among the up-regulated genes in UF NB-hop tumors, we also identified HIF-1a. An elevated HIF-1a expression fits with hypoxic, aggressively growing, and necrotic NB tumors, thus confirming that NB-hop is able to predict the hypoxic status of NB. 

We found distinct evidences of the association between hypoxia and TMM in NB. The enrichment of various pathways and processes that are involved in telomere maintenance as well as the up-regulation of TERT gene was, in fact, observed in the group of UF NB-hop tumors. The aberrant expression of TERT has been described to be closely associated with tumorigenesis [111], and the activation of telomerase through over-expression of TERT is thought to represent the most common pathway for cellular immortalization [25]. The expression of the TERT gene has been shown to correlate with telomerase activity in experiments involving NB tumor tissues [112] and to be a prognostic marker in various adult and pediatric tumors, including NB, where high levels of telomerase expression/activity were found to predict poor outcome [113,114]. Telomere lengthening to achieve immortalization is also obtained through ALT [24,115], a telomerase-independent mechanism that accounts for 10–15% of NB tumors that has been associated with unfavorable prognosis in NB [24]. We did not find an association between hypoxia and ALT mechanism for telomerase lengthening, while our findings support the conclusion that hypoxia induces telomere maintenance in NB through activation of telomerase-dependent mechanisms. 

Although telomerase regulation by hypoxia through the HIF-1α, the main regulator of gene transcription by hypoxia, has been described in both cancer and normal cells [116,117,118,119,120], the association between hypoxia and telomerase-dependent telomere maintenance has never been described in NB tumors. Thus, here we show, for the first time, that hypoxia may drive a novel potential telomerase-dependent mechanism for telomere maintenance in NB. 

More than 60% of NB tumors are metastatic, and secondary tumor sites can be found in bone, bone marrow, liver, lymph nodes, or, less commonly, in the skin, lung, or brain [121]. Metastasis is the main cause of NB-related deaths, making the investigation of the processes involved in tumor metastasization mandatory [122]. Cell adhesion is the process by which cells interact and attach to neighboring cells through specialized glycoproteins of the cell surface called cell adhesion molecules (CAMs) [123]. The loss of cell adhesion allows malignant cells to detach and escape from the primary mass gaining a more motile and invasive phenotype [124]. CAMs, such as cadherin, integrins, selectins, immunoglobulins, and CD44, are known to play a key role in each step of the metastasization process and are expressed in NB tumor cells [125]. Hypoxia promotes invasion and metastasization in NB by regulating CAMs expression [121]. Our data highlight a clear association between hypoxic tumors and metastatic disease (INSS stage 4), and provide evidences of the down-regulation of a large set of genes that are involved in cell adhesion within the group of UF NB-hop tumors. Furthermore, the loss of cell adhesion may also occur as a consequence of down-regulation of the CD44, CDH1, and L1CAM, which are well known CAMs in NB [76,78,81]. 

An important enhancer of cancer invasion and metastasis is epithelial-to-mesenchymal transition (EMT), a process that allows epithelial cells to adopt a more mesenchymal state with stem-like properties [123]. EMT involves expression modulation of several genes, including CHD1, which encodes E-cadherin, one of the most important markers of EMT in several types of cancer, including NB [126]. Other genes that are implicated in EMT in NB are TWIST1 [127,128], PLK4 [83], KRT19 [79], and ERBB3 [79]. In addition, the down-regulation of NRP1, CD9, and SRCIN1 encoding genes was reported to induce metastasis and invasion in NB [77,82,84]. Although hypoxia induces EMT in different types of cancer by modulating the expression of specific marker genes [129], the association between hypoxia and EMT in NB tumors has not been investigated. We found down-regulation of CDH1, CD9, SRCIN1, NRP1, KRT19, ERBB3 expression, and up-regulation of TWIST1 and PLK4 in UF NB-hop tumors. Furthermore, our data indicate the potential poor differentiation state of neurons associated with UF NB-hop tumors, as shown by the down-regulation of NTRK1, CAMTA1, DNER, and PTN, as well as the modulation of the known neuronal differentiation markers of NB RET, ALK, and PROM1 [67,68,69,70,72,74]. These data indicate that hypoxic NB tumors display gene expression modulation compatible with a mesenchymal state with stem-like properties and an increased invasiveness and metastasization potential.

Cellular proliferation is a hallmark of cancer [130]. A complex network of transcription factors, kinases, and cell cycle regulators is exploited by NB cells to proliferate [130]. Up to now, the mechanisms that mediate NB cell proliferation in response to hypoxia have not been fully elucidated [23]. In this study, we report that UF NB-hop tumors display the expression of genes that are involved in cellular proliferation, cell cycle, mitotic cell cycle, and cell division, among which we report AURKA, ERBB4, LIN28B, LMO3, MYCN, ODC1, and RAN genes, whose overexpression has been previously documented to induce proliferation in NB cells [50,51,52,53,54,55,56]. 

The abundance and composition of immune cells that infiltrate the TME is a critical determinant of tumor development, therapy efficacy, and clinical outcome in NB [22,97]. Cytotoxic T lymphocytes, B lymphocytes, and NK cells are responsible for the elimination of cancer cells [131,132,133,134]. Their massive infiltration in the TME is associated with a better clinical outcome of NB [22,135,136]. Monocytic-lineage cells are a heterogenous population of immune cells and a key component of the innate defense mechanisms that display a dual influence on tumor progression, having the potential to activate immunosurveillance and exert anti-tumor responses, but becoming subverted by the tumor to support its progression, spread, and immune evasion [137,138]. Myeloid dendritic cells (DCs) are professional antigen-presenting cells that are central to the orchestration of innate and acquired immunity which were described in the TME of many cancer types, and their inactivation was reported as one of the main mechanisms by which tumors can escape from immune surveillance [139,140]. Hypoxia is one of the critical signals conditioning the balance between immune cell anti-/pro-tumoral functions and it has been extensively reported to contribute to immune resistance and tumor escape mechanisms favoring an immunosuppressive TME in different types of cancer [141]. The mechanisms adopted by NB cells to evade immune response and grow uncontested have been extensively reviewed [142,143]. However, the association between hypoxia and immune suppression has never been investigated in NB tumors. The data reported in this study show, for the first time, the association between an immunosuppressive TME and hypoxic NB tumors. We demonstrated the down-regulation of a large number of genes involved in T cell activation, lymphocyte activation and immune response and the down-regulation of genes previously reported to induce immune suppression in NB, including CADM1, CCL19, CCL2, CCR7, CD226, and CXCL12 [58,59,60,63,65,66]. Furthermore, we found low abundance of infiltrating cytotoxic T and NK cells, DCs, and monocytic lineage cells in UF NB-hop tumors by MCP-counter analysis [90], highlighting the presence of immunosuppressive conditions in the hypoxic TME favorable for NB tumor growth and metastatic spread. These findings are in accordance with previous evidences reporting a low level of infiltrated lymphocytes, monocytes, and macrophages in the population of high-risk amplified MYCN primary metastatic NB tumors [97]. However, overlapping analysis between the HIF-1a gene network and MCP-counter markers did not show a direct interaction between HIF-1a and MCP-counter markers. Instead, network analysis based on public knowledge highlighted an indirect functional association between HIF-1a and MCP-counter markers. Transcriptional regulation by hypoxia is a complex process that involves several regulatory elements, including NF-kB, Ets-1, C/EBPα/β, AP-1, and Egr-1 transcription factors [144]. Therefore, not every effect of hypoxia is mediated by HIFs [144]. These considerations may potentially explain the lack of direct interaction between HIF-1a and MCP-counter markers. 

We decided to carry out a correlation analysis between HIF-1a and each MCP-counter marker in order to investigate this association in NB. Although network, correlation and MCP-counter analyses are based on different methodological and statistical approaches, results obtained with these methods support the association between immune suppression and hypoxic TME in NB. Because the tumor suppressing/promoting role of several immune markers remains controversial to date, we also assessed the potential suppressive role of the immune markers significantly correlated with HIF-1a. Bibliographic search evidenced that MAL, BANK1, CXCR2, and KCNJ15 genes play a tumor suppressive role in different types of cancer, including colorectal cancer, B-cell lymphoma, and renal cell carcinoma [93,94,95,96]. However, we did not find any study reporting the tumor suppressive role of these genes in NB. 

The high expression of TAM-specific genes was associated with poor five-year event-free survival in not amplified MYCN tumors [98]. In order to find additional associations between hypoxia and immune suppressive TME in NB, we correlated the expression of HIF-1a and that of prognostic TAM-specific genes. The significant correlation between the expression of HIF-1a and TAMs-specific genes supported the conclusion that hypoxia is an unfavorable prognostic marker and the potential association between immune suppression and a hypoxic TME in NB. These results are in agreement with previous findings that showed the association between cancer macrophage infiltration and immune suppression and highlighted the regulatory role of HIF-1a in cancer-associated macrophages polarization and infiltration [20]. 

Immune checkpoints are important pathways that are exploited by tumor cells to escape immune system response [99]. We analyzed the expression of B7-H3 and PD-Ls, immune checkpoints known to limit both the NK and T cell-mediated cytolytic activity, in order to assess the link between hypoxia and immune checkpoint expression in NB tumors. B7-H3 is highly expressed by NB and it is considered an unfavorable prognostic factor [99,145]. Our findings reported the up-regulation of B7-H3 in UF NB-hop tumors indicating a potential B7-H3-mediated immune regulation in this group of tumors. PD-L1 is up-regulated in NB by immunostimulatory cytokines, such as IFN-g [99], and the effect of its expression on patient survival is controversial [146]. Our results support an inverse correlation between PD-L1 expression and NB patient prognosis.

Despite the therapeutic advances in the last years, a large number of NB patients still undergo a refractory disease [147]. The mechanisms inducing resistance to therapy has gained great attention [147]. Apoptosis deregulation, the presence of cancer stem cells, alterations of drug target, augmented capacity of DNA repair, faster drug effluence, and autophagy have been reported as the main factors mediating NB cell acquisition of drug resistance [44]. However, little is known regarding the effects of hypoxia on these processes. Hypoxia has been associated with treatment resistance to a number of anticancer agents. Graham and Unger have documented the efficacy of twenty-two cancer drugs in human cell lines of non-pediatric cancers that were cultured in vitro under varying oxygen concentrations [18]. Five out of the six therapeutic agents conventionally used in the treatment of high-risk NB patients are among the cancer drugs whose efficacy was shown to be reduced by hypoxia [18]. Hypoxia promotes the NB chemoresistance to etoposide, vincristine, and cisplatin, which are three chemotherapeutic drugs used in the front-line treatment of high-risk NB patients [148,149]. Chemotherapeutic agents induce catastrophic DNA disruption with the consequent cancer cell death. However, DNA repair mechanisms are induced in cancer cells to overcome drugs-associated DNA damages [21]. In this study, we provide multiple evidences that UF NB-hop tumors up-regulate genes that are involved in different DNA damage response mechanisms, such as DNA repair, double-strand break repair, base-excision repair, and mismatch repair. Concordantly, we report the up-regulation of genes, including BRCA1, BRCA2, CHEK1, CHEK2, and TPX2, which play a regulatory role in the DNA damage response in NB [40,41,42,43,44].

p53 is a transcription factor that is involved in cellular defense against malignant transformation [48]. Hypoxia is a potent activator of p53 in different types of cancers [23]. However, conflicting evidences exist on the status of activation of p53 pathway signaling in NB cells [48]. Our findings highlight the up-regulation of genes that are involved in the p53 signaling pathway, p53-mediated cell cycle arrest, and cellular senescence, as well as the up-regulation of genes, including CDK1, CDK2, and TP53, whose overexpression indicates the activation of cell cycle arrest in NB [45,46,48].

Cell growth arrest promotes cellular resistance to apoptosis, which is associated with an unfavorable prognosis [150]. Our findings indicate that UF NB-hop tumors, with respect to F NB-hop tumors, down-regulate genes that are involved in cell death and up-regulate genes, including BIRC5 and TWIST1, whose anti-apoptotic role in NB cells has been previously documented [85,86,87]. 

Altogether, our data indicate that NB patients with hypoxic tumors are potentially more susceptible to acquire resistance to conventional chemotherapeutic drugs than patients with normoxic tumors. We believe that information derived from the assessment of the hypoxic status of the tumors may potentially be used for taking chemotherapeutic decisions in order to improve treatment response and reduce the side effects that are associated with aggressive therapies. 

Hypoxia is a potential therapeutic target and may offer several therapeutic opportunities in cancer treatment [18,151]. Therapeutic approaches targeting tumor hypoxia with hypoxia-activated prodrugs and topotecan [152] was shown to improve tumor response and prolong survival in NB xenograft models [153]. We believe that the combination of therapeutic agents targeting cancer cells and the TME counteracting the effects of inadequate tumor oxygenation could provide more effective anti-tumor immunity. The success of these new potential therapeutic advances depends on the assessment of the hypoxic status of the tumor at diagnosis. To this end, NB-hop may be instrumental to predict those NB patients who have a hypoxic tumor and may benefit from anti-hypoxia treatments. 

## 4. Materials and Methods 

### 4.1. Study Design and Patient Population

We used the gene expression profiles of 1882 primary tumor samples from four different cohorts of NB patients [6,7,8,9,10,11,12,13].

The gene expression profiles from 709 (Agilent709) and 498 (RNA-seq498) tumor samples were generated by Agilent customized 4 × 44 k oligonucleotide microarray [6] and Illumina HiSeq 2000 platform [7], respectively. All of the experimental and clinical data for Agilent709 and RNA-seq498 have been previously reported [7,8] and they are publicly accessible through the Gene Expression Omnibus (http://www.ncbi.nlm.nih.gov/geo; accession: gse62564) and ArrayExpress (http://www.ebi.ac.uk/arrayexpress; accession: E-MTAB-1781) databases. 

The third data set cohort (Affymetrix413) consisted of 413 NB patients that belonged to six sub-cohorts whose gene expression has been profiled using Affymetrix U133 Plus 2.0 GeneChips. Eighty-eight patients were collected by the Academic Medical Center (AMC; Amsterdam, The Netherlands) [9]; twenty-one patients were collected by the University Children’s Hospital, Essen, Germany and were treated according to the German NB trials; 51 patients were collected at the Hiroshima University Hospital or affiliated hospitals and were treated according to the Japanese NB protocols [10]; 173 patients were collected at the Gaslini Institute and were treated according to the Associazione Italiana Ematologia e Oncologia Pediatrica (AIEOP) or the International Society of Pediatric Oncology Europe NB (SIOPEN) protocols; 30 patients were collected from the United Kingdom [11]; and, another 50 patients from France [12]. For the Affymetrix413 cohort overall and event-free survivals were available for 291 patients. 

Gene expression profiles from additional 262 (Agilent262) tumor samples were generated by the Agilent customized 4 × 44 k oligonucleotide microarray [13]. Gene expression profiles were coupled with patient clinical and molecular data. Data were reported in the original manuscript [13], or are publicly accessible through the gene expression omnibus (GEO) at the accession GSE120572. 

The identifiers of the different data sets were converted into the corresponding gene symbols to make them comparable to each other. Probe sets with no gene symbol annotation were excluded from the analysis because they have no multi-platform annotation. Patients that were profiled with more than one platform were considered once in order to prevent that inclusion may introduce optimistic and biased predictions. 

Samples were obtained from primary tumors at the time of diagnosis. Tumor stage was defined according to the INSS stage. The data were retrieved from the R2: genomic analysis and visualization platform (http://r2.amc.nl), GEO or the BIT-Gaslini biobank. 

### 4.2. Outcomes

The primary endpoint was the OS defined as the time (in years) from disease diagnosis to patient death or the last follow-up if the patient survived. Alive patients with follow-up less than five-years were filtered out to make our primary endpoint as homogeneous as possible across patients. The secondary outcome was the EFS defined as the time (in years) from disease diagnosis to tumor progression, relapse, or death from disease or the last follow-up if no event occurred. 

### 4.3. Procedures

The COMBAT algorithm implemented in inSilicoMerging package [28] was used to remove batch effects and integrate the gene expression profile of the Agilent709, RNA-seq498, and Affymetrix413 data sets into one merged data set. PVCA was used in order to assess the magnitude of any source of variability [28]. The efficiency of batch effect removal process was measured by WAPV, and the batch effect was considered to be removed when the WAPV of the platform was 0.0. Splitting a data set into a training set and a test set is a standard machine learning procedure for computing an unbiased estimation of the performance of a classifier. The batch-adjusted data set was randomly split into a training set (30%) and a test set (70%). The Agilent262 data were split into a training set (21%) and a test set (79%). TMM and p53/RAS gene mutations data were arbitrarily included in the test set for assessing their association with hypoxia. The LibSVM library implementation for the support vector machines, included in the software WEKA 3.7.13 (University of Waikato, Hamilton, New Zealand), was used to build the classifier [154]. Multilayer perceptron, J48, Naïve Bayes, and linear regression algorithms implemented in the software WEKA 3.7.13 (University of Waikato, Hamilton, New Zealand) were used for performance comparison. LOOCV assessed a gene prognostic impact and the classifier performances. The classifier performances were estimated by Accuracy, Sensitivity, Precision, Specificity, Negative predictive value, and MCC measures. MCC was used as a reference measure for model selection. Fisher exact test was used to assess the significance of the association between classifier prediction and clinical outcome. The ConfusionMatrix function implemented in the Caret R Package [29] assessed the significance of the NB-hop classifier performance. Gene ontology (GO), REACTOME, and KEGG analyses were carried out on the list of up or down regulated genes in UF NB-hop tumors while using the STRING-DB software [89]. A FDR lower than 0.05 identified significantly enriched ontology terms and pathways. 

### 4.4. Statistical Analysis

OS and EFS curves were plotted by the Kaplan-Meier method and they were compared with the log-rank test by GraphPad Prism version 6.0 for Windows, www.graphpad.com. Exact log-rank test was provided when log-rank *p*-value was >0.0001. The univariate Cox model was used to assess the prognostic value of the markers when patient follow-up was available. Multivariate analysis with a Cox proportional hazards regression model was used to assess the prognostic effect of hypoxia signature in the context of concomitant effects of other known prognostic factors (i.e., age at diagnosis, INSS stage, and MYCN status). The patients with missing data were discarded from the survival analysis. The Survival R package (R 3.1.2) was used for the computation of the Cox regression models. Logistic regression analysis was carried out in order to assess the prognostic value of the markers when only event overall was available. Univariate logistic regression was computed using the bayesglm method implemented in the arm R package. Differential expression analysis considered as significant gene up- or down-regulation with fold change ≥1.5 or ≤1.5 (0.58 in log2) and *p* value lower than 0.05 after adjustment for multiple hypothesis testing by Benjamini–Hochberg method. We used the MCP-counter method in order to assess the abundance of different tumor-infiltrating immune and stromal cell populations [90]. MCP-counter defines a list of genes that are characteristic for each cell type and uses these markers to calculate a numeric abundance score for each immune cell population and non-immune stromal cell population [90]. Immune cell population involves T cells, CD8+ T cells, NK cells, cytotoxic lymphocytes, B cell lineage, monocytic lineage cells, myeloid dendritic cells, and neutrophils. The non-immune stromal populations include endothelial cells and fibroblasts [90]. BioGRID is a public curated gene interaction repository [92]. BioGRID was used to build gene interaction networks. Correlation was assessed by Pearson’s correlation coefficient. 

## 5. Conclusions

In this study, we reported independent evidences of the unfavorable prognostic value of hypoxia in NB and highlighted the role of hypoxia as a condition for the development and progression of NB. We identified NB-hop as a new biomarker that is able to define a new population of patients with hypoxic tumor and unfavorable prognosis, characterized by the activation of telomere maintenance mechanisms and a deregulated hypoxic TME. We believe that hypoxia evaluation should, thus, be considered for NB patient risk stratification and treatment. A prospective study and further in vitro and in vivo assessment of the effects of hypoxia in NB initiation and progression may guide the future development of hypoxia-directed therapeutic strategies. 

## Figures and Tables

**Table 1:** Platform information and clinical and biological characteristics of NB patients within the four cohorts used in the study.

Platform	RNA-seq498 (*n* = 498)	Affymetrix413 (*n* = 413)	Agilent709 (*n* = 709)	Agilent262 (*n* = 262)
**Name**	Illumina HiSeq 2000 RNASeq	Affymetrix HG-U133 plus 2.0	Agilent 44 K oligonucleotide array	Agilent 44 K oligonucleotide array
**Probe set Annotation**	RefSeq	Proprietary	Proprietary	Proprietary
**Number of Probe Sets**	43,827	40,352	43,290	43,290
**Patients’ Characteristics**				
**Age at Diagnosis**				
<18 months	300 (60.2%)	201 (48.6%)	431 (60.7%)	146 (55.8%)
≥18 months	198 (39.8%)	160 (38.7%)	276 (39.3%)	116 (44.2%)
na	0 (0%)	52 (12.7%)	0 (0%)	0 (0%)
**INSS Stage**				
1	121 (24.3%)	88 (21.3%)	158 (22.2%)	30 (11.5%)
2	78 (15.7%)	56 (13.5%)	116 (16.3%)	37 (14.1%)
3	63 (12.7%)	63 (15.2%)	92 (12.9%)	33 (12.6%)
4	183 (36.7%)	153 (37%)	259 (36.0%)	130 (49.6%)
4s	53 (10.6%)	44 (10.6%)	80 (11.2%)	32 (12.2%)
na	0 (0%)	9 (2.4%)	4 (1.4%)	0 (0%)
**MYCN Status**				
normal	401 (80.5%)	329 (79.6%)	581 (80.5%)	191 (72.9%)
amplified	92 (18.5%)	76 (18.4%)	122 (18.5%)	70 (26.7%)
na	5 (1.0%)	8 (2%)	6 (1.0%)	1 (0.4%)
**Event Overall**				
no	393 (78.9%)	282 (68.3%)	548 (77.3%)	202 (77.1%)
yes	105 (21.1%)	81 (19.6%)	161 (22.7%)	60 (22.9%)
na	0 (0%)	50 (12.1%)	0 (0%)	0 (0%)
**Event-free**				
no	315 (63.2%)	216 (52.3%)	439 (60.3%)	148 (56.5%)
yes	183 (36.8%)	96 (23.2%)	249 (36.8%)	114 (43.5%)
na	0 (0%)	101 (24.5%)	21 (2.9%)	0 (0%)
**Follow-up Duration (Years)**	5.4 (3.0–8.6)	2.1 (0.9–4.6)	5.6 (3.0–8.7)	na
**Telomere Maintenance**				
no	0 (0%)	0 (0%)	0 (0%)	99 (37.7%)
yes	0 (0%)	0 (0%)	0 (0%)	109 (41.6%)
na	262 (100%)	262 (100%)	262 (100%)	54 (20.7%)
**ALT**				
no	0 (0%)	0 (0%)	0 (0%)	177 (67.5%)
yes	0 (0%)	0 (0%)	0 (0%)	31 (11.8%)
na	262 (100%)	262 (100%)	262 (100%)	54 (20.7%)
**Documented Regression**				
no	0 (0%)	0 (0%)	0 (0%)	190 (72.5%)
yes	0 (0%)	0 (0%)	0 (0%)	18 (6.8%)
na	262 (100%)	262 (100%)	262 (100%)	54 (20.7%)
**TERT Rearrangements**				
no	0 (0%)	0 (0%)	0 (0%)	231 (88.1%)
yes	0 (0%)	0 (0%)	0 (0%)	31 (11.9%)
na	262 (100%)	262 (100%)	262 (100%)	
**ATRX Mutation**				
-	0 (0%)	0 (0%)	0 (0%)	75 (28.7%)
Deletion	0 (0%)	0 (0%)	0 (0%)	7 (2.8%)
Non sense	0 (0%)	0 (0%)	0 (0%)	1 (0.1%)
na	262 (100%)	262 (100%)	262 (100%)	179 (68.4%)
**RAS Mutations**				
no	0 (0%)	0 (0%)	0 (0%)	5 (1.9%)
yes	0 (0%)	0 (0%)	0 (0%)	43 (16.4%)
na	262 (100%)	262 (100%)	262 (100%)	214 (81.7%)
**RAS Mutations**				
no	0 (0%)	0 (0%)	0 (0%)	36 (13.7%)
yes	0 (0%)	0 (0%)	0 (0%)	12 (4.6%)
na	262 (100%)	262 (100%)	262 (100%)	214 (81.7%)

Data are relative to the patients within the four datasets RNA-seq498, Affymetrix413, Agilent498, and Agilent262 used in the study. In each subdivision, data show the total number of patients and the relative percentage within brackets. na indicates not available. NB: neuroblastoma. INSS: International Neuroblastoma Staging System. ALT: Alternative lengthening of telomere.

**Table 2:** NB-hop gene signature used in the prognostic model.

NB-hop	Gene Title	Affymetrix Probe Sets	Chromosome	Band	Hazard Ratio (95% CI)	*p* Value
**High Expression is Associated with Poor Prognosis**						
PGK1	phosphoglycerate kinase 1	200738_s_at, 17356_s_at	X	q21.1	6.5 (3.9–10.7)	**<0.0001**
PDK1	pyruvate dehydrogenase kinase, isozyme 1	206686_at, 226452_at	2	q31.1	1.8 (1.4–2.4)	**<0.0001**
MTFP1	mitochondrial fission process 1	223172_s_at	22	q12.2	2.4 (1.8–3.1)	**<0.0001**
FAM162A	family with sequence similarity 162, member A	223193_x_at	3	q21.1	1.9 (1.3–2.8)	**<0.0001**
EGLN1	egl nine homolog 1 (C. elegans)	224314_s_at	1	q42.2	1.0 (0.5–2.0)	>0.05
AK4	adenylate kinase 4	230630_at	1	p31.3	1.1 (0.7–1.6)	>0.05
**High Expression is Associated with Good Prognosis**						
ALDOC	aldolase C, fructose-bisphosphate	202022_at	17	q11.2	0.7 (0.5, 0.8)	**<0.001**

Univariate analysis was carried out by Cox regression using overall survival in the batch-adjusted training set. Significant *p*-values are depicted in bold. Genes with a hazard ratio greater than 1 were associated with poor prognosis. Genes with a hazard ratio smaller than 1 were associated with good prognosis. CI: confidence interval; NB: neuroblastoma.

**Table 3:** Univariate and multivariate analysis of overall and event-free survival in the batch-adjusted test set.

		Univariate Analysis			Multivariate Analysis	
Covariate	HR	95% CI	*p* Value	HR	95% CI	*p* Value
**Overall survival**						
NB-hop (UF vs. F)	5.4	(3.9–7.4)	**<2.0 × 10^−16^**	1.8	(1.2–2.6)	**4.20 × 10^−3^**
Age at diagnosis (≥18 months vs. <18 months)	7.4	(5.0–10.9)	**<2.0 × 10^−16^**	3.6	(2.3–5.5)	**8.50 × 10^−9^**
INSS stage (4 vs. 1, 2, 3, 4 s)	5.9	(4.0–8.5)	**<2.0 × 10^−16^**	2	(1.3–3.1)	**8.60 × 10^−4^**
MYCN status (Amplified vs. normal)	6.8	(5.0–9.4)	**<2.0 × 10^−16^**	2.7	(1.8–3.9)	**3.70 × 10^−7^**
**Event-free survival**						
NB-hop (UF vs. F)	3.4	(2.6–4.5)	**<2.0 × 10^−16^**	1.7	(1.2–2.5)	**1.40 × 10^−3^**
Age at diagnosis (≥18 months vs. <18 months)	3	(2.2–3.9)	**7.11 × 10^−15^**	1.8	(1.3–2.5)	**2.20 × 10^−4^**
INSS stage (4 vs. 1, 2, 3, 4 s)	2.9	(2.2–3.8)	**2.83 × 10^−14^**	1.6	(1.1–2.2)	**4.60 × 10^−3^**
MYCN status (Amplified vs. normal)	3.4	(2.6–4.5)	**<2.0 × 10^−16^**	1.6	(1.1–2.3)	**4.40 × 10^−3^**

Univariate and multivariate analysis of overall and event-free survival assessed by Cox regression in the test set. Significant p-values are depicted in bold. HR: Hazard ratio. CI: Confidence interval. UF: unfavorable. F: favorable. INSS: international neuroblastoma staging system.

**Table 4:** Summary of Kaplan-Meier estimates of OS and EFS based on the NB-hop prediction after subdividing patients of the batch-adjusted test set into sub-cohorts.

		F NB-hop			UF NB-hop		
Population	Survival Probability	SEM	No. of Patients	Survival Probability	SEM	No. of Patients	*p*
Stage 1 (*n* = 95)			91			4	
5-year OS	0.98	0.01		0.5	0.2		<0.0001
5-year EFS	0.95	0.02		0	0		<0.0001
Stage 2 (*n* = 85)			78			7	
5-year OS	0.96	0.02		0.57	0.18		<0.0001
5-year EFS	0.75	0.05		0.33	0.19		0.06
Stage 3 (*n* = 73)			56			17	
5-year OS	0.87	0.04		0.29	0.11		<0.0001
5-year EFS	0.65	0.06		0.23	0.1		0.008
Stage 4 (*n* = 231)			133			98	
5-year OS	0.63	0.04		0.32	0.04		<0.0001
5-year EFS	0.5	0.04		0.25	0.04		<0.0001
Stage 4s (*n* = 66)			56			10	
5-year OS	0.89	0.04		0.9	0.09		ns
5-year EFS	0.72	0.06		0.77	0.13		ns
Age at diagnosis <18 months (*n* = 321)			279			42	
5-year OS	0.94	0.01		0.61	0.07		<0.0001
5-year EFS	0.79	0.02		0.46	0.07		<0.0001
Age at diagnosis >=18 months (*n* = 229)			135			94	
5-year OS	0.62	0.04		0.27	0.04		<0.0001
5-year EFS	0.51	0.04		0.2	0.04		<0.0001
MYCN single copy (*n* = 436)			382			54	
5-year OS	0.88	0.01		0.5	0.06		<0.0001
5-year EFS	0.72	0.02		0.32	0.06		<0.0001
MYCN amplified (*n* = 111)			29			82	
5-year OS	0.31	0.08		0.3	0.05		ns
5-year EFS	0.33	0.09		0.25	0.04		ns

SEM indicates the standard error of mean. ns stands for not significant result. Survival curves have been compared by approximate log-rank test or exact log-rank test when approximate log-rank *p* value < 0.0001 or >0.0001, respectively.

**Table 5:** Summary of the biological processes and pathways significantly modulated in the UF respect to F NB-hop patients of the test set.

Biological Process ^a^	Gene Set Id ^b^	Pathway or Process Description ^c^	Number of Genes ^d^	FDR q-Value ^e^	Type of Regulation ^f^	Ontology ^g^
Response to hypoxia						
	GO: 0071456	Cellular response to hypoxia	19	1.00 × 10^−3^	UP	GO BP
	GO: 0001666	Response to hypoxia	26	2.00 × 10^−2^	UP	GO BP
Telomere Maintenance						
	HSA-180786	Extension of Telomeres	17	1.68 × 10^−10^	UP	RCTME
	HSA-157579	Telomere Maintenance	21	1.64 × 10^−9^	UP	RCTME
	HSA-174417	Telomere C-strand (Lagging Strand) Synthesis	13	5.90 × 10^−8^	UP	RCTME
	GO: 0000723	Telomere maintenance	22	1.00 × 10^−6^	UP	GO BP
	GO: 0032201	Telomere maintenance via semi-conservative replication	11	2.77 × 10^−6^	UP	GO BP
Chromatin remodeling						
	GO: 0031497	Chromatin assembly	31	9.97 × 10^−9^	UP	GO BP
	GO: 0031055	Chromatin remodeling at centromere	15	2.02 × 10^−6^	UP	GO BP
	GO: 0006325	Chromatin organization	66	3.32 × 10^−6^	UP	GO BP
	GO: 0006338	Chromatin remodeling	26	5.45 × 10^−6^	UP	GO BP
DNA damage response						
	GO: 0051276	Chromosome organization	143	3.32 × 10^−27^	UP	GO BP
	GO: 0007059	Chromosome segregation	67	5.21 × 10^−24^	UP	GO BP
	HSA-73886	Chromosome Maintenance	32	1.86 × 10^−14^	UP	RCTME
	GO: 0006281	DNA repair	77	4.27 × 10^−16^	UP	GO BP
	HSA-73894	DNA Repair	47	9.85 × 10^−11^	UP	RCTME
	GO: 0006302	Double-strand break repair	29	1.88 × 10^−6^	UP	GO BP
	hsa03410	Base excision repair	10	4.00 × 10^−4^	UP	KEGG
	GO: 0006284	Base-excision repair	9	5.00 × 10^−3^	UP	GO BP
	hsa03430	Mismatch repair	6	2.00 × 10^−2^	UP	KEGG
P53 mediated Cell cycle arrest and cellular senescence						
	HSA-6791312	TP53 Regulates Transcription of Cell Cycle Genes	14	6.62 × 10^−6^	UP	RCTME
	HSA-6804756	Regulation of TP53 Activity through Phosphorylation	18	1.99 × 10^−5^	UP	RCTME
	GO:0006977	DNA damage response, signal transduction by p53 class mediator resulting in cell cycle arrest	14	1.00 × 10^−4^	UP	GO BP
	HSA-6804116	TP53 Regulates Transcription of Genes Involved in G1 Cell Cycle Arrest	7	2.40 × 10^−4^	UP	RCTME
	HSA-6804114	TP53 Regulates Transcription of Genes Involved in G2 Cell Cycle Arrest	7	7.50 × 10^−4^	UP	RCTME
	hsa04218	Cellular senescence	20	2.00 × 10^−3^	UP	KEGG
	hsa04115	p53 signaling pathway	12	4.00 × 10^−3^	UP	KEGG
Cell cycle and proliferation						
	GO: 0007049	Cell cycle	196	6.38 × 10^−43^	UP	GO BP
	GO: 0000278	Mitotic cell cycle	136	4.17 × 10^−42^	UP	KEGG
	GO: 0051301	Cell division	87	1.15 × 10^−21^	UP	GO BP
	GO: 0008283	Cell population proliferation	64	1.07 × 10^−5^	UP	GO BP
Immune response						
	GO: 0046649	Lymphocyte activation	63	1.87 × 10^−7^	DOWN	GO BP
	GO: 0042110	T cell activation	45	1.27 × 10^−6^	DOWN	GO BP
	GO: 0002250	Immune response	168	7.98 × 10^−6^	DOWN	GO BP
Cell differentiation						
	GO: 0030154	Cell differentiation	365	2.59× 10^−11^	DOWN	GO BP
	GO: 0022008	Neurogenesis	193	6.90 × 10^−11^	DOWN	GO BP
	GO: 0030182	Neuron differentiation	134	3.75 × 10^−10^	DOWN	GO BP
	GO: 0002521	Leukocyte differentiation	51	2.60 × 10^−5^	DOWN	GO BP
	GO: 0030217	T cell differentiation	29	4.39 × 10^−5^	DOWN	GO BP
	GO: 0030098	Lymphocyte differentiation	39	1.30 × 10^−4^	DOWN	GO BP
Cell motility						
	GO: 0048870	Cell motility	108	7.41 × 10^−5^	DOWN	GO BP
	HSA-1474244	Extracellular matrix organization	45	1.00 × 10^−3^	DOWN	RCTME
	GO: 0007155	Cell adhesion	141	5.55 × 10^−15^	DOWN	GO BP
	hsa04514	Cell adhesion molecules (CAMs)	40	3.14 × 10^−9^	DOWN	KEGG
Inflammation response						
	GO: 0006954	Inflammatory response	71	6.87 × 10^−6^	DOWN	GO BP
	GO: 0071345	Cellular response to cytokine stimulus	111	9.18 × 10^−5^	DOWN	GO BP
	GO: 0050727	Regulation of inflammatory response	44	6.70 × 10^−3^	DOWN	GO BP
	hsa04062	Chemokine signaling pathway	24	2.00 × 10^−2^	DOWN	KEGG
Cell death						
	GO: 0042981	Regulation of apoptotic process	153	7.90 × 10^−4^	DOWN	GO BP
	GO: 0043067	Regulation of programmed cell death	154	0.00086	DOWN	GO BP
Angiogenesis						
	GO: 0001568	Blood vessel development	61	0.00065	DOWN	GO BP
	GO: 0001525	Angiogenesis	42	2.30 × 10^−3^	DOWN	GO BP
	GO: 0045766	Positive regulation of angiogenesis	25	1.20 × 10^−2^	DOWN	GO BP

^a^ Significant GO biological processes, Reactome terms, and KEGG pathways. GO, Reactome and KEGG enrichment analysis was carried out on genes whose expression in UF NB-hop compared with F NB-hop prediction was significantly modulated. ^b^ Official identifier of a GO biological process, Reactome or KEGG pathway. ^c^ Official name of a GO biological process, Reactome term or KEGG pathway. ^d^ Number of genes of a GO biological process or Reactome term or KEGG pathway whose expression was significantly modulated in UF NB-hop tumors. ^e^ FDR q-value estimates the significance of the enrichment of a biological process or a pathway. FDR q-value <= 0.05 are considered acceptable. ^f^ Type of regulation of the genes involved in a process or a pathway. ^g^ Name of the ontology defining a biological process or pathway. GO BP stands for gene ontology biological process. KEGG stands for Kyoto Encyclopedia of Genes and Genomes. RCTME stands for Reactome.

**Table 6:** Summary of selected genes whose modulation was significant in the UF NB-hop patients.

Biological Process ^a^	Gene Symbol ^b^	Gene Name ^c^	Locus ^d^	OMIM ^e^	Type of Regulation ^f^	Function ^g^	References ^h^
Response to hypoxia							
	GAPDH	glyceraldehyde-3-phosphate dehydrogenase	12p13.31	138400	UP	GAPDH is a gene encoding a key enzyme in glycolysis and it is a hypoxia-induced gene in NB.	[32]
	HK2	hexokinase 2	2p12	601125	UP	HK2 is a gene encoding a protein that plays a key role in maintaining the integrity of the outer mitochondrial membrane. HK2 is a hypoxia-induced gene in NB and its up-regulation was associated with unfavorable prognosis in NB.	[3]
	LDHA	lactate dehydrogenase A	11p15.1	150000	UP	LDHA is a gene encoding an important component of the lactate dehydrogenase tetramer enzyme crucial for aerobic glycolysis. Increased expression of LDHA is associated with decreased survival and aggressive disease in NB including amplification of MYCN, older age, stage 4 and undifferentiated histology.	[33]
	LDHB	lactate dehydrogenase B	12p12.1	150100	UP	LDHB is a gene encoding an important component of the lactate dehydrogenase tetramer enzyme crucial for aerobic glycolysis. LDHB contributes to aggressiveness of NB.	[33]
	PDK1	pyruvate dehydrogenase kinase 1	2q31.1	602524	UP	PDK1 is a gene encoding a mitochondrial multienzyme complex that catalyzes the oxidative decarboxylation of pyruvate. PGK1 is a hypoxia-induced gene in NB and its up-regulation was associated with unfavorable prognosis in NB.	[5]
	PGK1	phosphoglycerate kinase 1	Xq21.1	311800	UP	PGK1 is a gene encoding an enzyme that catalyzes one of the two ATP producing reactions in the glycolytic pathway. PGK1 is a hypoxia-induced gene in NB and its up-regulation was associated with unfavorable prognosis in NB.	[5]
	PKM	pyruvate kinase M1/2	15q23	179050	UP	PKM encodes a protein involved in glycolysis and lactate production. PKM2 expression is elevated in high stage NB.	[34]
	SLC16A1	solute carrier family 16 member 1	1p13.2	600682	UP	SLC16A1 gene encodes a proton-linked monocarboxylate transporter that catalyzes the movement of several monocarboxylates including lactate and pyruvate across the plasma membrane. High SLC16A1 mRNA expression is significantly associated with worse prognosis in NB.	[35]
	SLCO4A1	solute carrier organic anion transporter family member 4A1	20q13.33	612436	UP	SLCO4A1 is a gene encoding a membrane transporter of which the only currently known solute is thyroid hormone. SLCO4A1 is a hypoxia-induced gene and its up-regulation was associated with unfavorable prognosis in NB.	[3]
Telomere Mantainance							
	FEN1	flap structure-specific endonuclease 1	11q12.2	600393	UP	FEN1 is a DNA repair and replication endonuclease and exonuclease that has been shown to play a critical role for maintaining genomic integrity. FEN1 is a potent MYCN target gene in NB.	[36]
	PCNA	proliferating cell nuclear antigen	20p12.3	176740	UP	PCNA is a gene coding a protein that acts as the initial sensor of telomere damage. PCNA levels are increased in tumors with an amplified N-myc gene and in metastatic stage tumors in NB.	[37]
	TERT	telomerase reverse transcriptase	5p15.33	187270	UP	TERT is a gene encoding a key component of the telomerase complex. TERT plays a key role in telomere maintenance in NB. High expression levels of TERT is an unfavorable prognostic markers in NB.	[13]
Chromatin remodeling							
	ARID1A	AT-rich interaction domain 1A	1p36.11	603024	DOWN	ARID1A is a chromatin-remodeling gene required for transcriptional activation of genes. ARID1A is a tumor suppressor gene in NB. ARID1A gene knockdown promotes NB migration and invasion.	[38]
	CHD5	chromodomain helicase DNA binding protein 5	1p36.31	610771	DOWN	CHD5 is a chromatin-remodeling gene that maps to 1p36.31 and is a tumor suppressor gene in NB. Low or absent *CHD5* expression is associated with a 1p36 deletion and an unfavorable outcome in NB.	[39]
DNA damage response							
	BRCA1	BRCA1 DNA repair associated	17q21.31	113705	UP	BRCA1 is a gene encoding a nuclear phosphoprotein. BRCA1 protein keeps NB cells alive by cooperating with MYCN. BRCA1 expression closely correlated with MYCN amplification and was a strong indicator of poor prognosis in NB.	[40]
	BRCA2	BRCA2 DNA repair associated	13q13.1	600185	UP	BRCA2 is a gene encoding a protein involved in double-strand break repair and/or homologous recombination. BRCA2 is one of the genes for which somatic mutations have been identified in primary NB.	[41]
	CHEK1	checkpoint kinase 1	11q24.2	603078	UP	CHK1 is a protein-coding gene belonging to serine/threonine-protein kinase. CHK1 performs a central role in DNA damage response and in preserving genomic integrity. CHK1 overexpression is thought to contribute to NB aggressiveness and poor NB patient survival.	[42]
	CHEK2	checkpoint kinase 2	22q12.1	604373	UP	CHK2 is a protein-coding gene belonging to serine/threonine-protein kinase. CHK2 plays a role in DNA damage response and maintenance of chromosomal stability. CHK2 overexpression is thought to contribute to NB aggressiveness.	[43]
	TPX2	TPX2 microtubule nucleation factor	20q11.21	605917	UP	TPX2 is a protein-coding gene involved in spindle apparatus assembly. TPX2 plays a principal function in the DNA damage response pathway. High TPX2 expression is significantly associated with poor prognosis in NB patients.	[44]
P53 mediated Cell cycle arrest and cellular senescence							
	CDK1	cyclin dependent kinase 1	10q21.2	116940	UP	CDK1 is a gene encoding a protein involved in the control of the eukaryotic cell cycle. CDK1 plays an essential role in NB tumor cell survival. CDK1 overexpression is associated with low survival for NB patients independently from MYCN status.	[45]
	CDK2	cyclin dependent kinase 2	12q13.2	116953	UP	CDK2 is gene encoding a member of a family of serine/threonine protein kinases that participate in cell cycle regulation. High CDK2 expression is strongly correlated with a bad prognosis.	[46]
	TP53	tumor protein p53	17p13.1	191170	UP	TP53 is a gene encoding a transcription factor that plays a critical role in the cellular defense against malignant transformation by promoting cell-cycle arrest, DNA damage repair, apoptosis, and senescence in response to stress signals. TP53 is a hypoxia-inducible gene.	[47,48]
Cell cycle and proliferation							
	AURKA	aurora kinase A	20q13.2	603072	UP	AURKA is a gene encoding a member of a family of mitotic serine/threonine kinases. AURKA is a critical regulator of hypoxia-mediated tumor progression in NB. Overexpression of AURKA has been associated with poor prognosis in NB.	[49,50]
	ERBB4	erb-b2 receptor tyrosine kinase 4	2q34	600543	UP	ERBB4 gene encodes a member of the Tyr protein kinase family and the epidermal growth factor receptor subfamily. HER4 functions as a cell cycle suppressor, maintaining resistance to cellular stress in NB. High HER4 expression significantly correlates with reduced survival in NB.	[51]
	LIN28B	lin-28 homolog B	6q16.3–q21	611044	UP	LIN28B is an RNA binding protein that blocks the maturation of let-7. LIN28B is an oncogene in NB. High LIN28B expression induces an increased cell proliferation. High LIN28B expression is associated with poor NB patient survival.	[52]
	LMO3	LIM domain only 3	12p12.3	180386	UP	LMO3 encodes a protein that belongs to the rhombotin family of cysteine-rich LIM domain oncogenes. LMO3 acts as an oncogene in NB. LMO3 induces marked tumor growth in nude mice. Increased expression of LMO3 is significantly associated with a poor prognosis in NB.	[53]
	MYCN	MYCN proto-oncogene, bHLH transcription factor	2p24.3	164840	UP	MYCN is one of the most important oncogenes in NB. MYCN plays multiple roles in malignancy and maintenance of stem-like state. Amplification of MYCN correlates with high-risk disease and poor prognosis in NB.	[54]
	ODC1	ornithine decarboxylase 1	2p25.1	165640	UP	ODC1 is a bona fide oncogene that encodes the rate-limiting enzyme in polyamine synthesis. Up-regulation of ODC1 induces a rapid tumor cell proliferation in NB. Elevated ODC1 is associated with reduced survival of NB patients.	[55]
	RAN	RAN, member RAS oncogene family	12q24.33	601179	UP	RAN encodes a small GTP binding protein belonging to the RAS superfamily. RAN promotes cell proliferation in neuroblastoma. High RAN expression is associated with a lower NB patient survival.	[56]
Immune response							
	CADM1	cell adhesion molecule 1	11q23.3	605686	DOWN	CADM1 encodes a protein that mediates homophilic cell-cell adhesion in a Ca(2+)-independent manner. CADM1 down-regulation is associated with unfavorable prognosis in NB. Inhibition of CADM1 in tumor cells enables immune evasion and promotes metastasis.	[57,58]
	CCL19	C-C motif chemokine ligand 19	9p13.3	602227	DOWN	CCL12 is a cytokine involved in immunoregulatory and inflammatory processes. NB induces profound functional impairments in CCR7/CCL19-mediated dendritic cell migration in vitro and in vivo.	[59]
	CCL2	C-C motif chemokine ligand 2	17q12	158105	DOWN	CCL2 is an immunoregulatory chemokine. MYCN represses expression of CCL2 inhibiting natural killer T cell chemoattraction in NB.	[60]
	CCR7	C-C motif chemokine receptor 7	17q21.2	600242	DOWN	CCR7 encodes a member of the G protein-coupled receptor family. Murine NB inhibits mature dendritic cell function decreasing antitumor immunity via down-regulating CCR7 expression. CXCR7 expression was low in undifferentiated NB tumors.	[61,62,63]
	CD226	CD226 molecule	18q22.2	605397	DOWN	CD226 encodes a glycoprotein expressed by virtually all human NK cells, T cells, and monocytes.	[64,65]
	CXCL12	C-X-C motif chemokine ligand 12	10q11.21	600835	DOWN	CXCL12 encodes a stromal cell-derived alpha chemokine member of the intercrine familyCXCL12 down-regulation in bone marrow samples from NB patients was strongly associated with worse EFS and OS.	[66]
	PVR	PVR cell adhesion molecule	19q13.31	173850	UP	PVR encodes a transmembrane glycoprotein that is widely expressed on normal neuronal, epithelial, endothelial, and fibroblastic cells and at high density on tumors of different histotype.	[64,65]
Cell differentiation							
	ALK	ALK receptor tyrosine kinase	2p23.2–p23.1	105590	UP	ALK is a receptor tyrosine kinase involved in neuronal differentiation in NB. High ALK expression correlates with an adverse NB phenotype. ALK is an oncogene in NB.	[67]
	CAMTA1	calmodulin binding transcription activator 1	1p36.31–p36.23	611501	DOWN	CAMTA1 encodes a transcriptional activator protein. Low CAMTA1 expression inhibits neuroblastoma cell differentiation. Low CAMTA1 expression is significantly associated with markers of unfavorable tumor biology and poor outcome in NB.	[68]
	DNER	delta/notch like EGF repeat containing	2q36.3	607299	DOWN	DNER is a protein involved in the activation of the NOTCH1 pathway. DNER is a marker of neural differentiation. Low DNER expression is associated with a low differentiation in NB.	[69]
	NTRK1	neurotrophic receptor tyrosine kinase 1	1q23.1	191315	DOWN	NTRK1 encodes a member of the neurotrophic tyrosine kinase receptor family. Low NTRK1 expression is associated with a poorly neuronal differentiated state and a significant worse outcome in NB.	[70,71]
	PROM1	prominin 1	4p15.32	604365	UP	PROM1 encodes a pentaspan transmembrane glycoprotein. PROM1 represses NB cell differentiation and is decreased by several differentiation stimulators. High PROM1 expression was significantly associated with a decreased probability of patient survival in NB.	[72,73]
	PTN	pleiotrophin	7q33	162095	DOWN	PTN encodes a secreted heparin-binding growth factor. PTN is a marker of neural differentiation. Low PTN expression is associated with a lower patient survival in NB.	[69,74]
	RET	ret proto-oncogene	10q11.21	164761	UP	RET is an oncogenic receptor tyrosine kinase involved in NB cell proliferation and differentiation. High expression of RET correlates with poor outcomes in patients with NB.	[71,75]
Cell motility and invasiveness							
	CD44	CD44 molecule (Indian blood group)	11p13	107269	DOWN	CD44 encodes a cell-surface glycoprotein involved in cell-cell interactions, cell adhesion and migration Lack of CD44 expression has been associated with MYCN amplification and predicts risk of disease progression and dissemination in NB.	[76]
	CD9	CD9 molecule	12p13.31	143030	DOWN	CD9 encodes a member of tetraspanin family. Low CD9 expression enhances inhibited migration and invasion in NB cells. Low *CD9* expression in primary neuroblastomas correlates with a low probability of patient survival in NB.	[77]
	CDH1	cadherin 1	16q22.1	192090	DOWN	The CDH1 gene encodes the epithelial cell adhesion molecule, which forms the core of the adherence junctions between adjacent epithelial cells. Low expression levels of CDH1 promote NB cell migration and invasion and poor patient survival.	[78]
	ERBB3	erb-b2 receptor tyrosine kinase 3	12q13.2	190151	DOWN	ERBB3 encodes a member of the epidermal growth factor receptor family of receptor tyrosine kinases. The decreased expression of ERBB3 was highly correlated with invasiveness in NB cell lines. The NB patients with low expression of ERBB3 showed significantly worse overall survival.	[79]
	L1CAM	L1 cell adhesion molecule	Xq28	308840	DOWN	L1CAM is a gene encoding a protein of the immunoglobulin superfamily of cell adhesion molecules. L1CAM plays a key role in the development of the nervous system. Low L1CAM expression is associated with a worse survival in NB patients.	[80,81]
	NRP1	neuropilin 1	10p11.22	602069	DOWN	NRP1 encodes a transmembrane glycoprotein. NB cells in which NRP1 was knocked down exhibited increased migratory and invasive abilities. Lower levels of NRP1 expression were significantly associated with a shorter survival period of patients with NB.	[82]
	PLK4	polo like kinase 4	4q28.1	605031	UP	PLK4 is one of the polo-like kinase family members. Up-regulation of PLK4 in NB cells induces EMT through the PI3K/Akt signaling pathway. High expression of PLK4 was negatively correlated with clinical features and survival.	[83]
	SRCIN1	SRC kinase signaling inhibitor 1	17q12	610786	DOWN	SRCIN1 is protein-coding gene that acts as a negative regulator of SRC. Low SRCIN1 expression correlates with increased metastatic recurrences in NB patients. Low SRCIN1 expression is associated with a poor prognosis in NB.	[84]
Cell death							
	BIRC5	baculoviral IAP repeat containing 5	17q25.3	603352	UP	BIRC5 is a member of the inhibitor of apoptosis gene family. The over-expression of BIRC5 correlates with an unfavorable prognosis in NB.	[85]
	MYC	MYC proto-oncogene, bHLH transcription factor	8q24.21	190080	DOWN	MYC encodes a nuclear phosphoprotein that plays a role in cell cycle progression, apoptosis and cellular transformation. MYC is a proto-oncogene in NB. MYC and MYCN expression is inversely correlated in primary NB.	[86]
	TWIST1	twist family bHLH transcription factor 1	7p21.1	601622	UP	TWIST1 encodes a basic helix–loop–helix–ZIP transcription factor with crucial functions during embryogenesis. TWIST1 plays a crucial role in inhibition of apoptosis and differentiation in NB.	[87]
Angiogenesis							
	EPAS1	endothelial PAS domain protein 1	2p21	603349	DOWN	EPAS1 encodes a transcription factor, which plays a key role during chronic hypoxia. Low level of EPAS1 expression is associated with higher NB patient survival.	[88]

^a^ Main biological function associated with listed genes. ^b^ Official gene identifier sorted in alphabetic order within each functional group. ^c^ Official gene name. ^d^ Position of the gene in the chromosome. ^e^ OMIM identifier of the gene. ^f^ Type of gene regulation in the population of patients with UF NB-hop patients. Type of regulation was determined by differential expression analysis. ^g^ Short description of the gene function as reported in the literature on NB. ^h^ Main references reporting gene function in NB.

**Table 7:** Univariate logistic regression analysis of known covariates on the Agilent262 test set.

Covariate	OR	95% CI	*p* Value
Age at diagnosis (≥18 months vs. <18 months)	3.1	(1.6–5.7)	**3.03 × 10^−4^**
ALT (Yes vs. No)	0.6	(0.2–1.5)	3.00 × 10^−1^
Event Overall (Yes vs. No)	8.4	(3.9–18.4)	**7.77 × 10^−8^**
Event-free (Yes vs. No)	4.3	(2.3–7.8)	**2.12 × 10^−6^**
TERT Rearrangement (Yes vs. No)	1.8	(0.7–4.4)	1.00 × 10^−1^
INSS stage (4 vs. 1, 2, 3, 4 s)	3.8	(2.1–6.9)	**1.11 × 10^−5^**
MYCN status (Amplified vs. Normal)	31.9	(13.2–73.9)	**1.21 × 10^−14^**
P53/RAS gene mutations (Yes vs. No)	3.7	(1.9–7.3)	**8.18 × 10^−5^**
Spontaneous regression (Yes vs. No)	0.1	(0.02–0.7)	**2.00 × 10^−2^**
Telomere.Maintenance (Yes vs. No)	16.70	(7.3–38.2)	**2.80 × 10^−11^**

Analysis was assessed by Logistic regression analysis using BayesGLM method. Covariates are sorted in alphabetic order. NB-hop prediction was used as reference class for the analysis. Significant *p*-values are depicted in bold. Odd ratio indicates the constant effect of hypoxia on the likelihood that one outcome will occur. OR: Odd ratio. CI: Confidence interval. UF: unfavorable. F: favorable. INSS: international neuroblastoma staging system; ALT: Alternative lengthening of telomeres.

## Supplementary Materials

The following are available online at https://www.mdpi.com/2072-6694/12/9/2343/s1, Table S1: Gene expression profile of 786 NB tumors after COMBAT batch-effect removal; Table S2: Clinical and biological characteristics of NB patients included in batch-adjusted training and test sets; Table S3: Clinical and molecular characteristics of NB patients in the batch-adjusted data set used in the present study; Table S4: Performance comparison among alternative machine learning methods on the batch-adjusted test set; Table S5: Differentially modulated genes in the batch-adjusted test set; Table S6: Significant biological processes enriched in the group of patients with NB-hop unfavorable using the batch-adjusted test set; Table S7: Correlation between HIF1a and MCP-counter markers in the batch-adjusted test set; Table S8: Correlation between HIF-1a and TAM-specific genes in the batch-adjusted test set without MYCN amplification; Table S9: Agilent262 data set used in the present study; Figure S1: PVCA results based on the gene expression profile of 786 NB tumors not adjusted for batch effect removal; Figure S2: PVCA results based on the gene expression profile of 786 NB tumors adjusted for batch effect removal; Figure S3: Distribution of HIF1a and EPAS1/HIF2a mRNA expression grouped by NB-hop prediction in the batch-adjusted test set; Figure S4: Network analysis among DEGs reported in Table 6 and HIF1a; Figure S5: HIF1a interaction network from BioGRID repository; Figure S6: Network analysis among HIF1a and MCP-counter markers; Figure S7: Distribution of CD276/B7-H3 and CD274/PD-L1 mRNA expression grouped by NB-hop prediction in the batch-adjusted test set.
