# Supplementary material for: Hypoxia Predicts Poor Prognosis in Neuroblastoma Patients and Associates with Biological Mechanisms Involved in Telomerase Activation and Tumor Microenvironment Reprogramming
Source: Cancers (Basel). 2020 Aug 19;12(9):2343. doi: 10.3390/cancers12092343 (PMC7563184; doi:10.3390/cancers12092343)
Supplement: Supplementary file 1 [file cancers-12-02343-s001.zip › cancers-852053 supplementray layout/cancers-852053-supplementary Figure S1-S7.docx]

Supplementary Materials

Hypoxia Predicts Poor Prognosis in Neuroblastoma Patients and Associates with Biological Mechanisms Involved in Telomerase Activation and Tumor Microenvironment Reprogramming

Davide Cangelosi, Martina Morini, Nicolò Zanardi, Angela Rita Sementa, Marco Muselli, Massimo Conte, Alberto Garaventa, Ulrich Pfeffer, Maria Carla Bosco, Luigi Varesio and Alessandra Eva


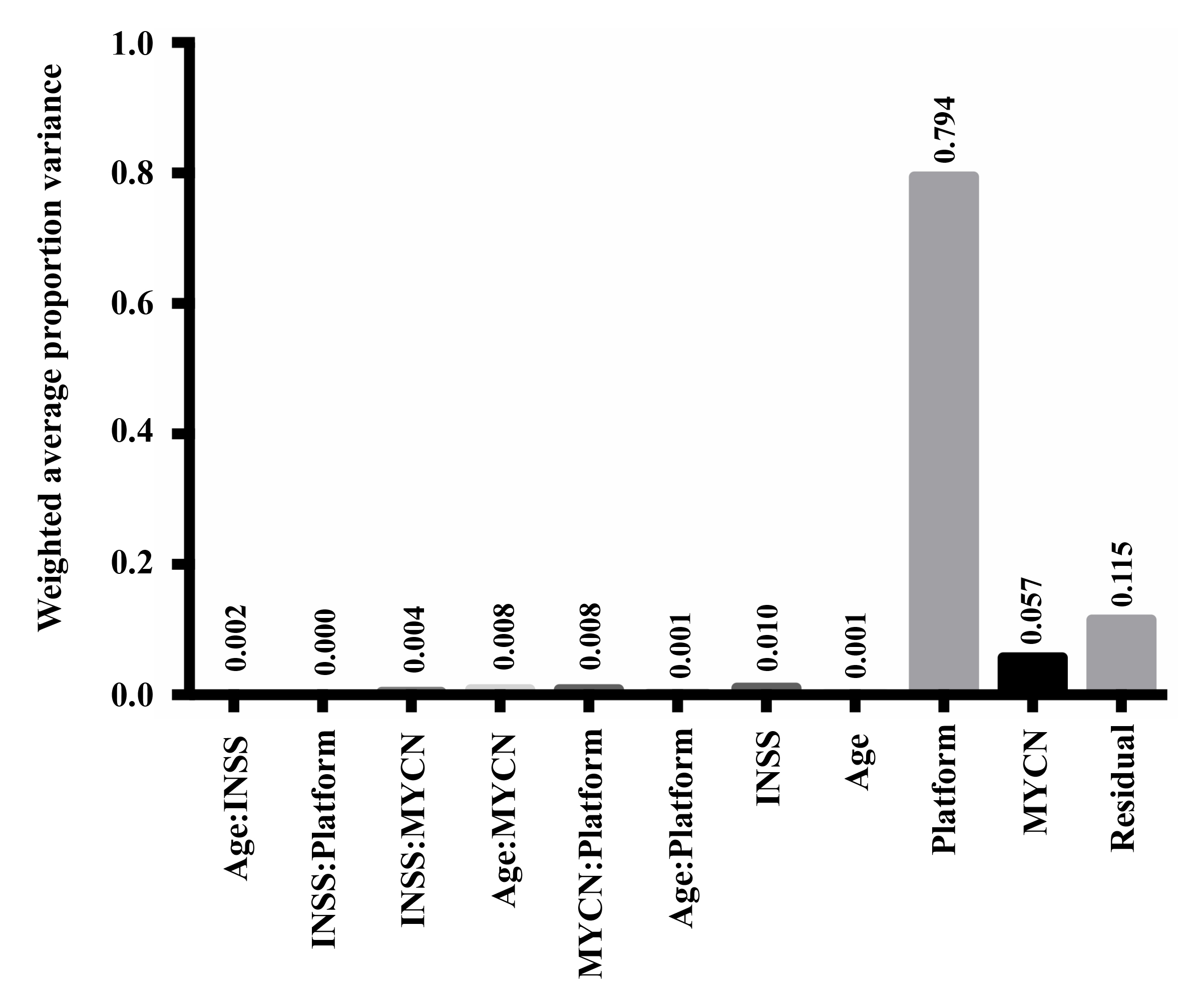


**Figure S1**. PVCA results based on the gene expression profile of 786 NB tumors not adjusted for batch effect removal. The plot shows the contribution of each factor to the overall variation estimated by PVCA. The variables used in the analysis are: Platform, age group, INSS stage and MYCN status. Platform was the reference variable for assessing the presence of batch effect. The bar plot displays the WAPV in the y-axis and the variables in the x-axis. The WAPV of each variable is also displayed on top of the bars. Interaction variables are labeled by the name of the two variables separated by colon. INSS: international neuroblastoma staging system.

**
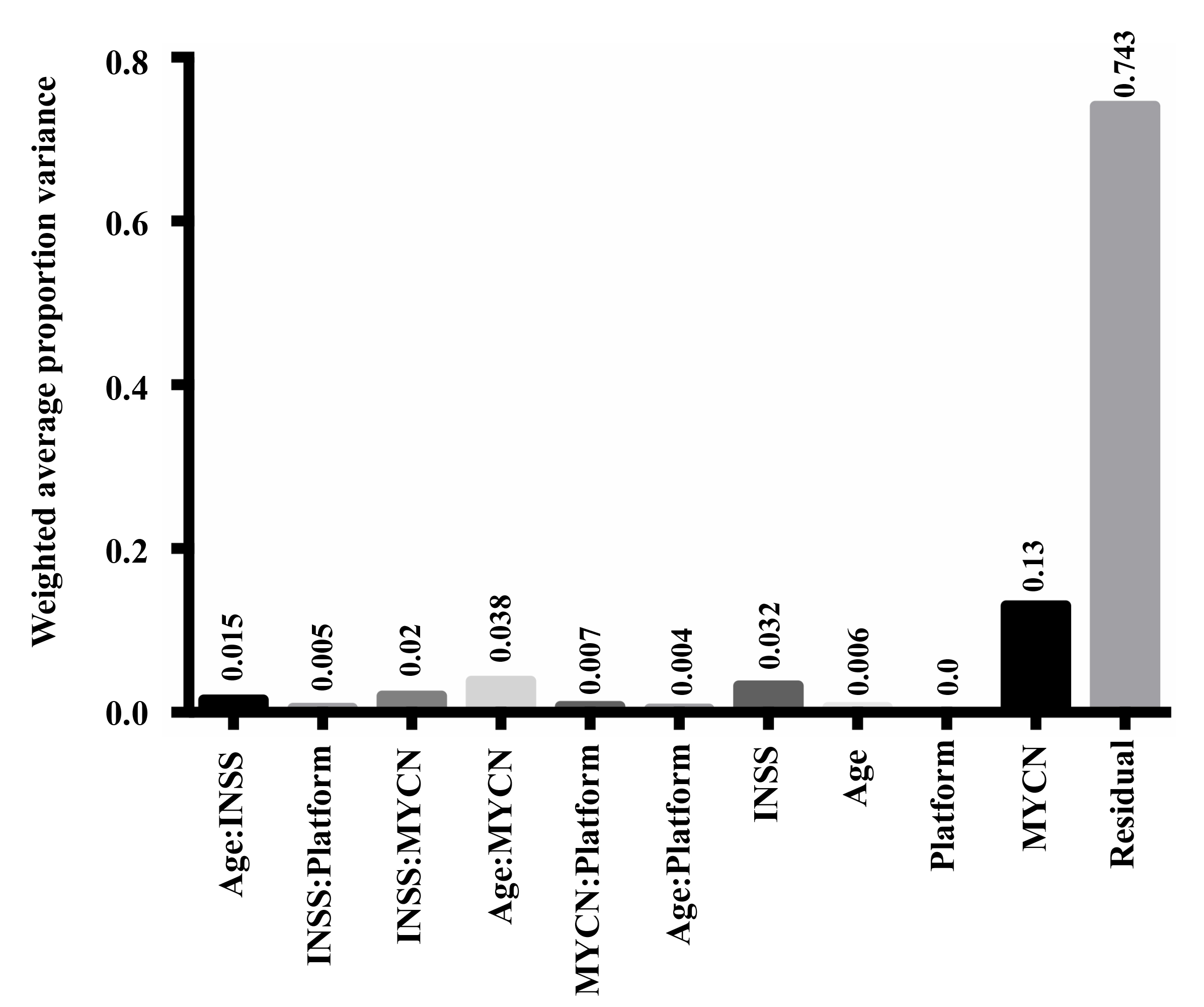
**

**Figure S2.** PVCA results based on the gene expression profile of 786 NB tumors adjusted for batch effect removal. The plot shows the contribution of each factor to the overall variation estimated by PVCA. COMBAT method was used to adjust the expression data among the RNA-seq498, Agilent709, and Affymetrix413 data sets. The variables used in the analysis are: Platform, age group, INSS stage and MYCN status. Platform was the reference variable for assessing the presence of batch effect. The bar plot displays the WAPV in the y-axis and the variables in the x-axis. The WAPV of each variable is also displayed on top of the bars. Interaction variables are labeled by the name of the two variables separated by colon. INSS: international neuroblastoma staging system.

**
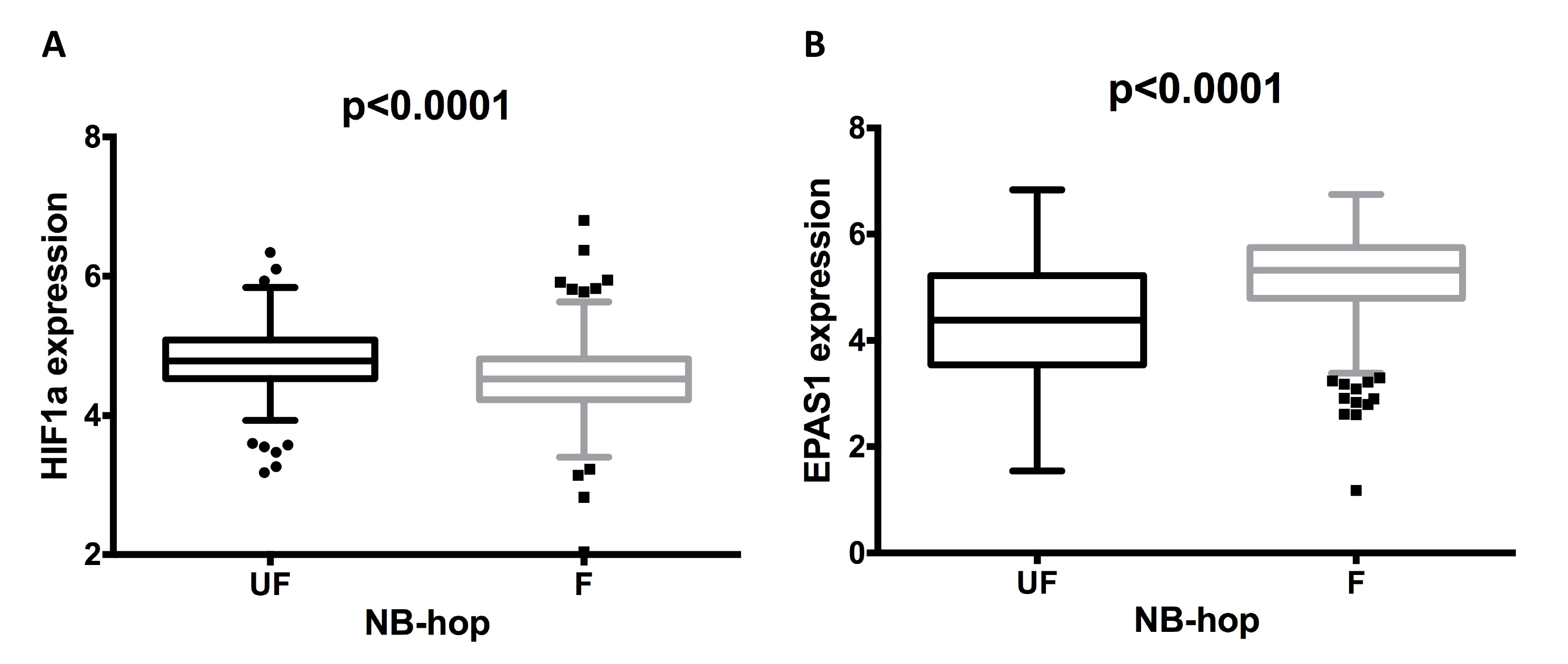
**

**Figure S3.** Distribution of HIF1a and EPAS1\HIF2a mRNA expression grouped by NB-hop prediction in the batch-adjusted test set. Box plot show the distribution of gene expression in HIF1a (panel A) and EPAS1/HIF2a (panel B) markers of NB patients grouped by NB-hop prediction. Data are relative to batch-adjusted test set (n = 550). Significance of the expression differences between F and UF NB-hop groups of patients was assessed by unpaired t test. *P*-value is reported on the top. F: Favorable; UF: Unfavorable; NB: Neuroblastoma.


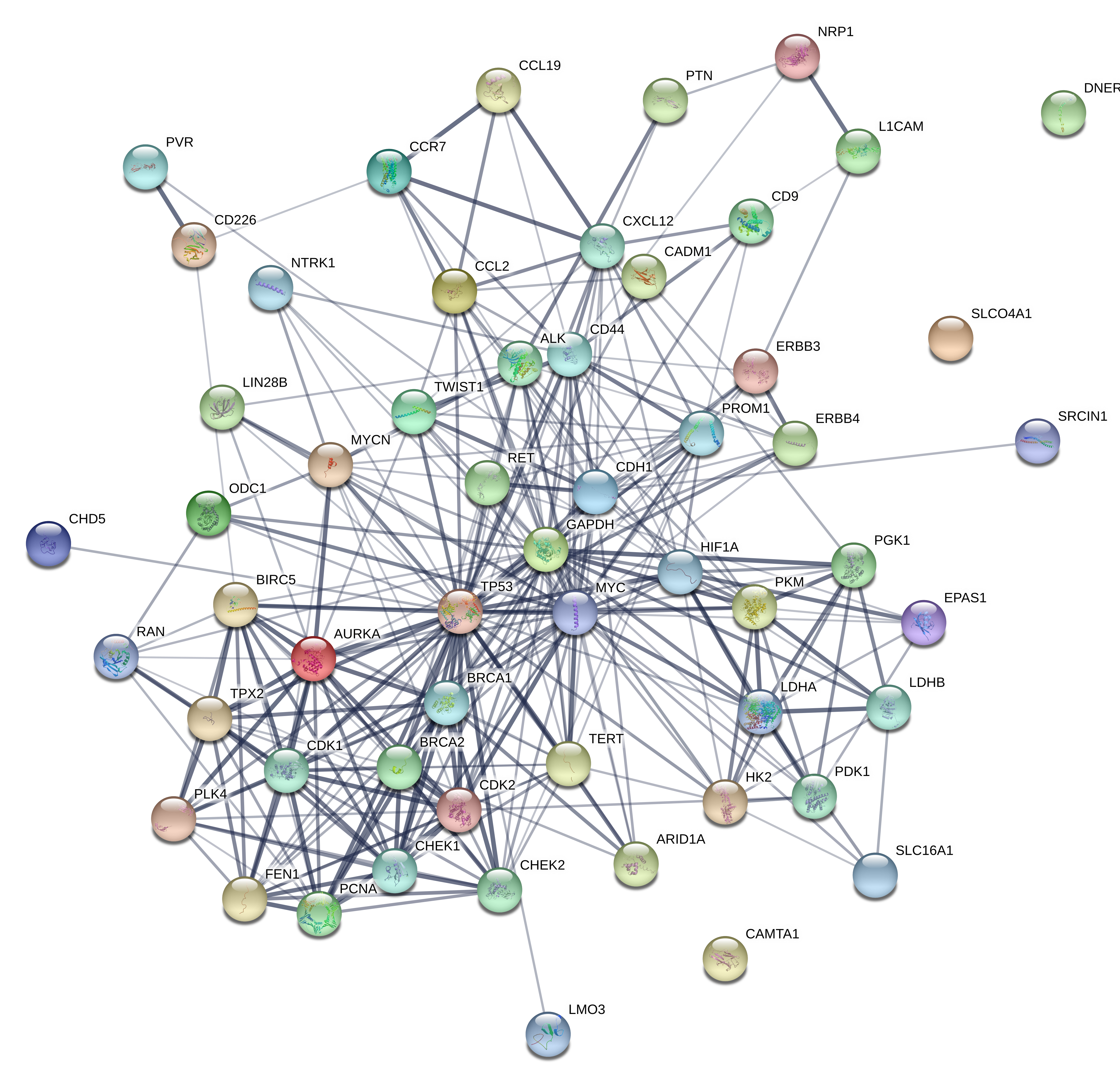


**Figure S4.** Network analysis among DEGs reported in Table 6 and HIF1a. The STRING-DB software was used to construct interaction networks among genes. Networks are displayed graphically as nodes (DEGs products) and edges (protein–protein association). The thickness of the line indicates the degree of confidence prediction of the association. Only associations with a high degree of confidence (0.4) are displayed in the plot.DEG: Differentially expressed genes.

**
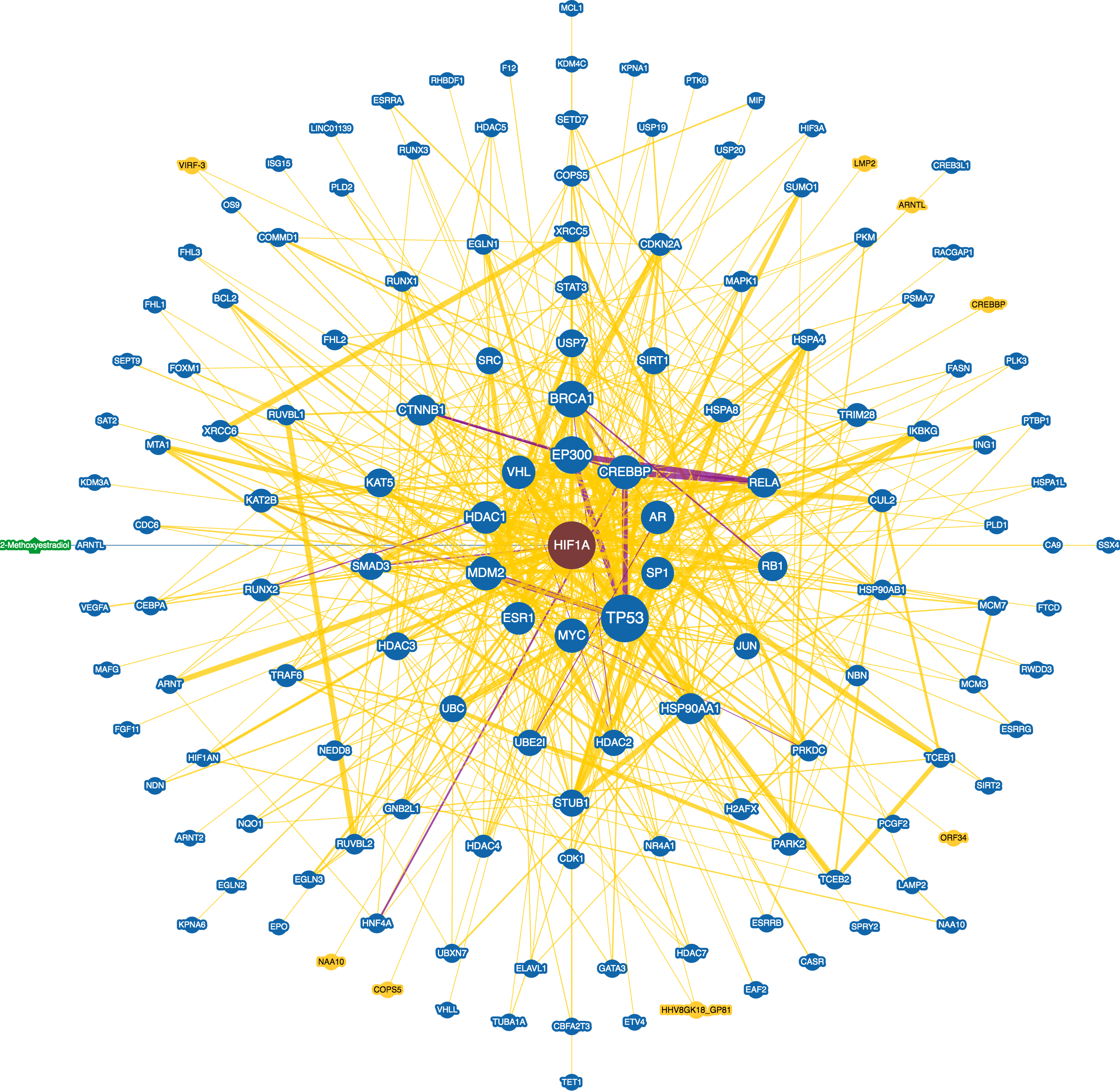
**

**Figure 5.** HIF1a interaction network from BioGRID repository. The network displays a network of colored nodes and edges. Each node identifies an interactor gene and the color indicates the reference gene (Brown), or if the associated gene is from the same (Blue) or different (Yellow) organism. Each edge between two nodes defines the type and strength of the evidence of interaction. Yellow edges indicates an association with physical evidence. Violet edges indicates an association with genetic and physical evidence. Greater node size represents increased connectivity and thicker edge sizes represent increased evidence supporting the association.


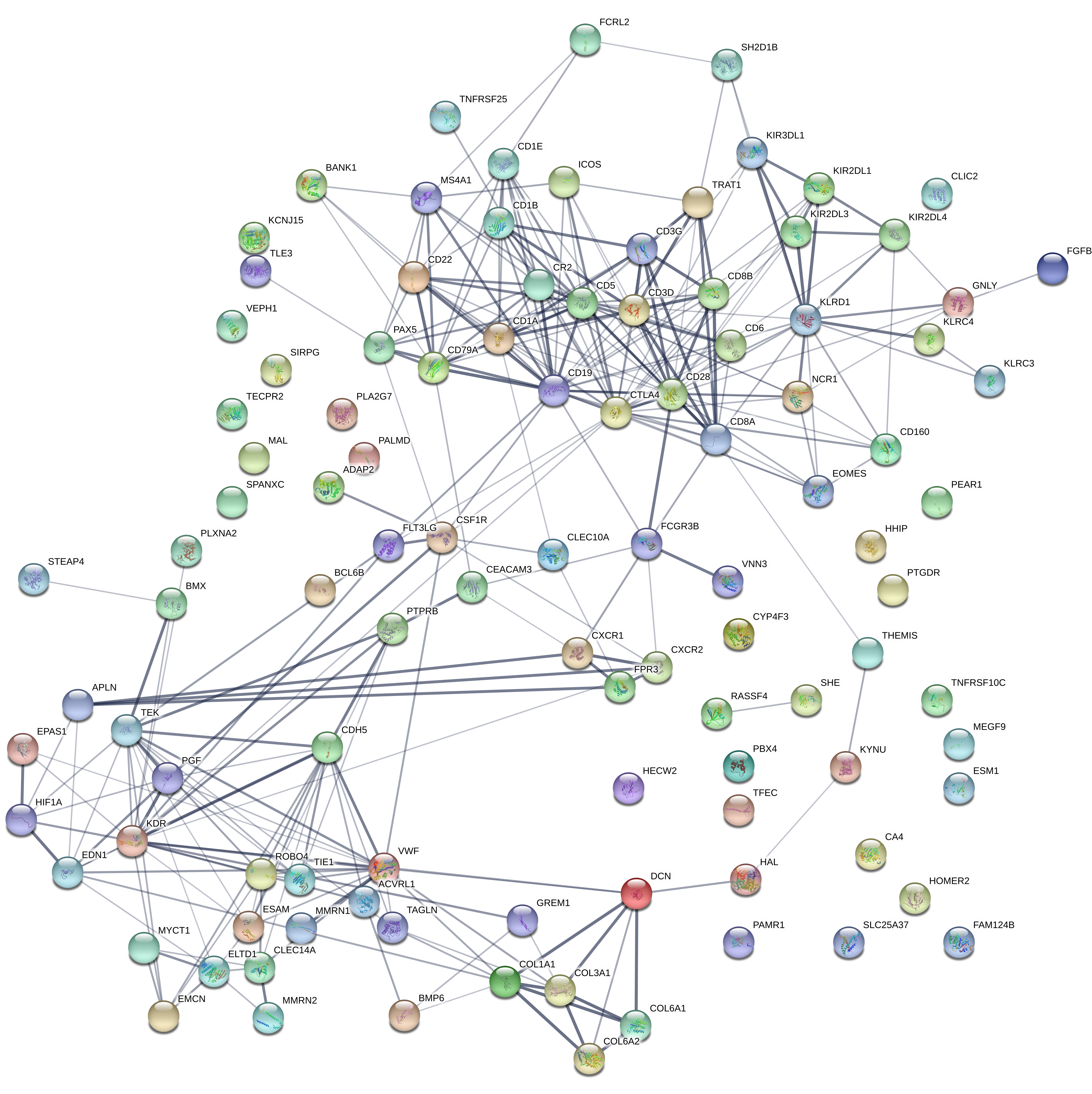


**Figure S6.** Network analysis among HIF1a and MCP-counter markers. The STRING-DB software was used to construct interaction networks among genes. Networks are displayed graphically as nodes (gene products) and edges (protein–protein association). The thickness of the line indicates the degree of confidence prediction of the association. Only associations with a high degree of confidence (0.4) are displayed in the plot. MCP: Microenvironment Cell Populations.


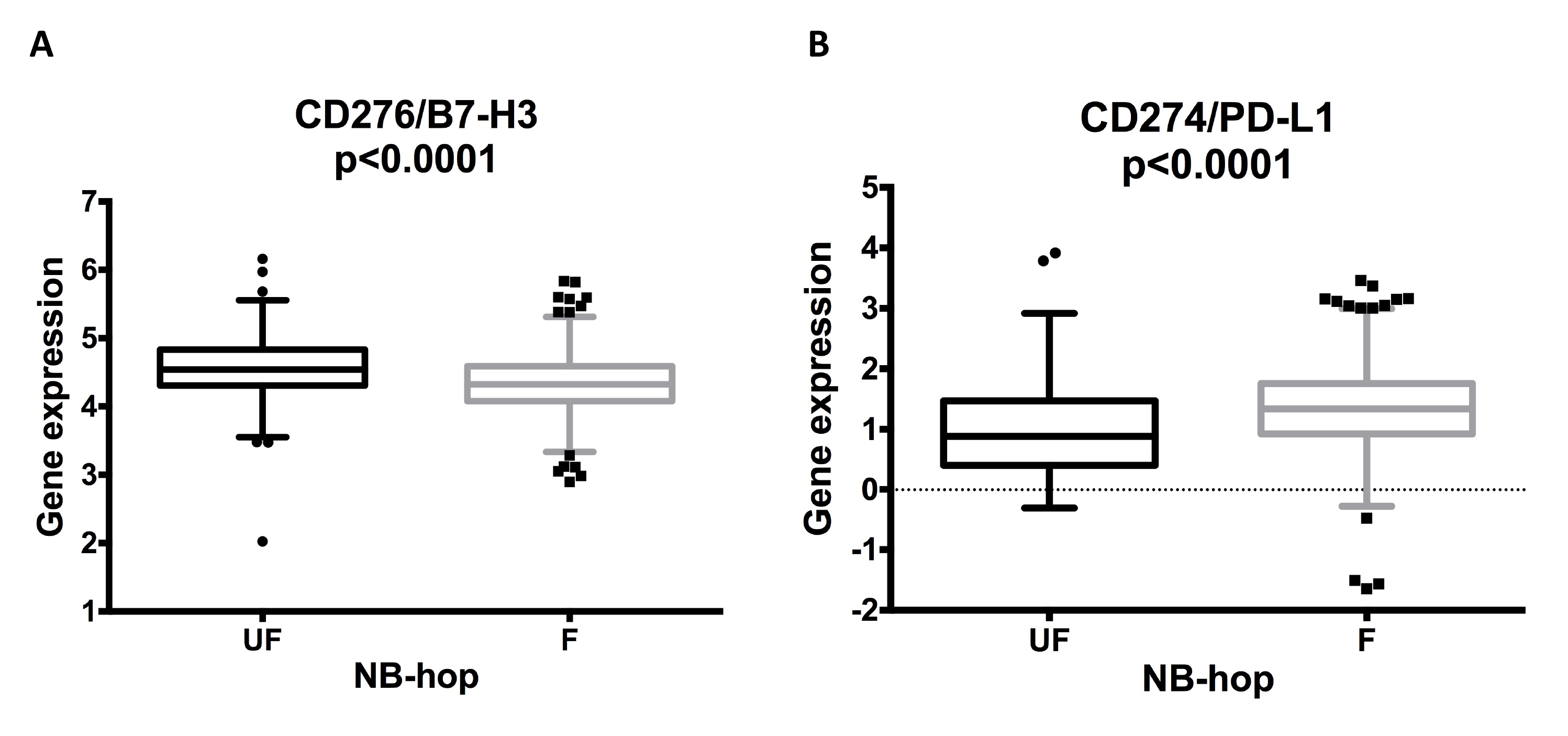


**Figure S7.** Distribution of CD276/B7-H3 and CD274/PD-L1 mRNA expression grouped by NB-hop prediction in the batch-adjusted test set. Box plot show the distribution of gene expression in CD276/B7-H3 (panel A), and CD274/B7-H1 (panel B) markers of NB patients grouped by NB-hop prediction. Data are relative to batch-adjusted test set (n = 550). Significance of the expression differences between F and UF NB-hop groups of patients was assessed by unpaired t test. P-value is reported on the top. F: Favorable; UF: Unfavorable; NB: Neuroblastoma.

| 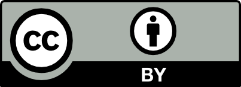 | © 2020 by the authors. Licensee MDPI, Basel, Switzerland. This article is an open access article distributed under the terms and conditions of the Creative Commons Attribution (CC BY) license (http://creativecommons.org/licenses/by/4.0/). |
| --- | --- |
